# Cochlear Implantation in Children with Additional Disabilities: A Systematic Review

**DOI:** 10.3390/children10101653

**Published:** 2023-10-05

**Authors:** Valeria Caragli, Daniele Monzani, Elisabetta Genovese, Silvia Palma, Antonio M. Persico

**Affiliations:** 1 Otorhinolaryngology-Head and Neck Surgery, Audiology Program, Department of Diagnostic Clinical and Public Health, University of Modena and Reggio Emilia, 41125 Modena, Italy; 196382@studenti.unimore.it; 2 Department of Surgery Dentistry Paediatrics and Gynaecology, University of Verona, 37100 Verona, Italy; daniele.monzani@univr.it; 3 Audiology Program, Department of Diagnostic Clinical and Public Health, University of Modena and Reggio Emilia, 41100 Modena, Italy; elisabetta.genovese@unimore.it; 4 Audiology, Primary Care Department, AUSL Modena, 41100 Modena, Italy; si.palma@ausl.mo.it; 5 Child and Adolescent Neuropsychiatry Program, Department of Biomedical, Metabolic and Neural Sciences, University of Modena and Reggio Emilia, Modena University Hospital, 41125 Modena, Italy

**Keywords:** autism, cerebral palsy, cochlear implant, intellectual disability, Usher syndrome, Waardenburg syndrome

## Abstract

This study examines the last 10 years of medical literature on the benefits of cochlear implantation in children who are deaf or hard of hearing (DHH) with additional disabilities. The most recent literature concerning cochlear implants (CIs) in DHH children with additional disabilities was systematically explored through PubMed, Embase, Scopus, PsycINFO, and Web of Science from January 2012 to July 2023. Our two-stage search strategy selected a total of 61 articles concerning CI implantation in children with several forms of additional disabilities: autism spectrum disorder, cerebral palsy, visual impairment, motor disorders, developmental delay, genetic syndromes, and intellectual disability. Overall, many children with additional disabilities benefit from CIs by acquiring greater environmental sound awareness. This, in turn, improves non-verbal communication and adaptive skills, with greater possibilities to relate to others and to be connected with the environment. Instead, despite some improvement, expressive language tends to develop more slowly and to a lesser extent compared to children affected by hearing loss only. Further studies are needed to better appreciate the specificities of each single disability and to personalize interventions, not restricting the analysis to auditory and language skills, but rather applying or developing cross-culturally validated instruments able to reliably assess the developmental trajectory and the quality of life of DHH children with additional disabilities before and after CI.

## 1. Introduction

Congenital hearing loss is one of the most frequent chronic conditions in children. The estimated prevalence of bilateral sensorineural hearing loss in infants is 1.3 cases per 1000 live births [1]. Conventional hearing aids can provide satisfactory rehabilitation opportunities for many patients with mild-to-moderate hearing loss. However, these technologies do not provide children with severe-to-profound sensorineural hearing loss with adequate sound clarity for them to understand speech. In recent decades, cochlear implants (CI) have become the preferred treatment for these patients.

A CI is an electronic device surgically placed under the skin in the mastoid region of the temporal bone and attached to an electrode array placed in the inner ear. The electrode array substitutes the activity of defective or absent cochlear hair cells by directly stimulating auditory nerve fibers. Sounds from the surrounding space are collected by the external sound processor, which contains a microphone, battery, and digital signal processing unit. The functional skills of the auditory system are highly dependent on the age at which deafness begins, and treatment with CI at younger ages usually provides the best outcome [2]. Early restoration of hearing function can reverse or rearrange some neurocognitive and neurological effects of sensory loss. In fact, stimulation with CI during the critical period in early development induces the maturation of auditory circuits, and at least partly compensates for these deficits in children due to brain plasticity [3].

About 20–40% of children born with severe-to-profound hearing deficits also have significant additional disabilities, which might, per se, prevent them from reaching a level of language, cognitive, or socio-communicative development comparable to DHH children without additional disabilities [4]. Additional disabilities in children typically include developmental delays, intellectual disabilities, visual impairments, cerebral palsy, autism spectrum disorder (ASD), attention deficit/hyperactivity disorder (ADHD), and motor disorders, among others.

In the past, comorbidity between deafness and behavioral, psychological, and cognitive disabilities was considered a contraindication for pediatric CI, under the assumption that the auditory information conveyed by the CI would not have carried the same meaning for children with additional disabilities as for those without additional disabilities [5]. In other words, these children would not have been able to process and analyze auditory information as efficiently as a child with hearing loss as the only disability [6]. On the one hand, the coincidence of one or more disabilities at the beginning of life in DHH children indeed generates a more complex condition, which requires a multidisciplinary approach for medical management. In addition, these children represent a challenge with regard to defining the most appropriate auditory amplification [7]. On the other hand, the frequent enrollment of very young children whose additional disabilities were diagnosed after surgery (as often occurs with ASD, intellectual disability, mild cerebral palsy, and progressive forms of sensory/cognitive deficits), advances in CI technology and a progressive broadening of indications for cochlear implantation have unveiled clear benefits for children with additional disabilities treated with CI, albeit with great interindividual variability [8].

The literature provides some evidence suggesting that children with additional disabilities may display some improvement in communication skills following cochlear implantation, despite the fact that auditory and speech outcomes largely depend on the severity of comorbid disorders and that many children with additional disabilities do not develop any intelligible speech at all [7,9,10]. On the other hand, the CI results in functional abilities in children with additional disabilities are less evident and unequivocal [11,12]. For example, children with autism have been proposed to generally have a more negative outcome based on some studies [13], but the evidence is mixed and requires some interpretation given the great heterogeneity of ASD (see Results and Discussion below). These results may not be entirely contradictory, because even if children with additional disabilities may not benefit from CI based on traditional outcome targets (auditory perception, receptive and expressive language), they have been reported to improve in terms of environmental awareness and quality of life [9].

In order to thoroughly assess the currently available evidence and, if possible, to provide clinicians with some evidence-based indications, the present study systematically reviews the outcome of cochlear implantation in DHH children with additional disabilities in terms of auditory perception, speech production, adaptive skills, and quality of life. To this aim, the last 10 years of medical literature have been searched by applying a two-stage search strategy, which has yielded 61 articles distinguished into four quality levels based on experimental design. These articles span a broad array of additional disabilities, including autism spectrum disorder, cerebral palsy, visual impairment, motor disorders, developmental delay, genetic syndromes, and intellectual disabilities.

## 2. Materials and Methods

### 2.1. Literature Search Strategy

A literature search of English-language studies was performed through PubMed, Embase, Scopus, PsycINFO, and Web of Science, according to the Preferred Reporting Items for Systematic reviews and Meta-Analysis (PRISMA) 2020 Statement guidelines [14]. The literature search was performed in two stages, always filtering for “English language”, “Humans”, and age 0–18 years (children and adolescents), with the years 2012–2023 selected:

(a) Stage 1—a broad search on all “additional disabilities” using the following string in “All Fields”:

(((((benefits) AND (cochlear implants) AND (((multiple)) AND (additional disabilities) OR (needs))))))

Based on stage 1, it was possible to identify single additional disabilities, namely, autism spectrum disorder (ASD), cerebral palsy, visual impairment, motor disorders, developmental delay, genetic syndromes, and intellectual disability.

(b) Stage 2—a targeted search was subsequently performed for each additional disability using the following strings:

(cochlear implant) AND (autism)

(cochlear implant) AND (cerebral palsy)

(cochlear implant) AND (visual impairment)

(cochlear implant) AND (motor disorders)

(cochlear implant) AND (intellectual disability)

(cochlear implant) AND (genetic syndrome)

(cochlear implant) AND (developmental delay)

Some studies included other disorders among “additional disabilities”, namely “ADHD”, “epilepsy”, “behavioral problems” and “medical disorders”. These terms were not included in our systematic search as independent entities, because: (a) The diagnostic threshold for “ADHD” can largely differ in different nations and medical communities and this can be predicted to enhance inter-study variability and to reduce data consistency. (b) “Epilepsy” cannot be assumed to necessarily represent an additional disability, because its forms differ widely and the evolution toward epileptic encephalopathy depends on the semiology and frequency of seizures, age at onset, drug response, and need for polypharmacy. Finally, (c) “behavioral problems” and “medical disorders” are too broad and non-specific.

This systematic review was not registered. The last literature search was performed on 4 July 2023. Once the original publications were collected, their bibliographies were manually searched for additional references.

### 2.2. Study Selection and Classification Criteria

The study selection process outlined in the previous paragraph was aimed at finding all the articles that were relevant to determining outcomes for DHH children with additional disabilities receiving a CI.

Studies were excluded if: (a) they contained duplicated data from other published work; (b) they were reviews, meta-analyses, or case reports; (c) they did not distinguish cochlear implant (CI) from hearing aid (HA) intervention when analyzing treatment outcomes; (d) they did not include DHH children with additional disabilities and/or did not specify the additional disability diagnosis; or (e) they enrolled children with normal hearing not treated with CI as controls (the appropriate control for CI-treated children with additional disabilities is CI-treated children without additional disabilities).

Finally, studies were divided into four classes based on the experimental design and the quality of the evidence (Table 1). In particular, studies were classified based on whether they recruited only cases or also controls, and whether they contrasted pre- to post-CI behavioral symptoms and quality of life or only collected post-CI data (Table 1).

### 2.3. Data Extraction

Following study selection, the data were independently extracted by two authors (V.C., S.P.) and reviewed by another author (A.M.P.). A consensus was reached through discussion if there was any disagreement. For every article selected in class 1, we collected the following information:

- Type of additional disability;
- N of cases (children with additional disability receiving CI);
- N of controls (children without additional disability receiving CI);
- Mean age (range) at cochlear implantation;
- The psychodiagnostic instruments used for auditory perception, language assessment, cognitive level, quality of life, etc.;
- Pre-implant differences between cases and controls;
- Post-implant outcomes.

All available information was collected for articles falling into classes 2–4.

## 3. Results

**Figure 1:**
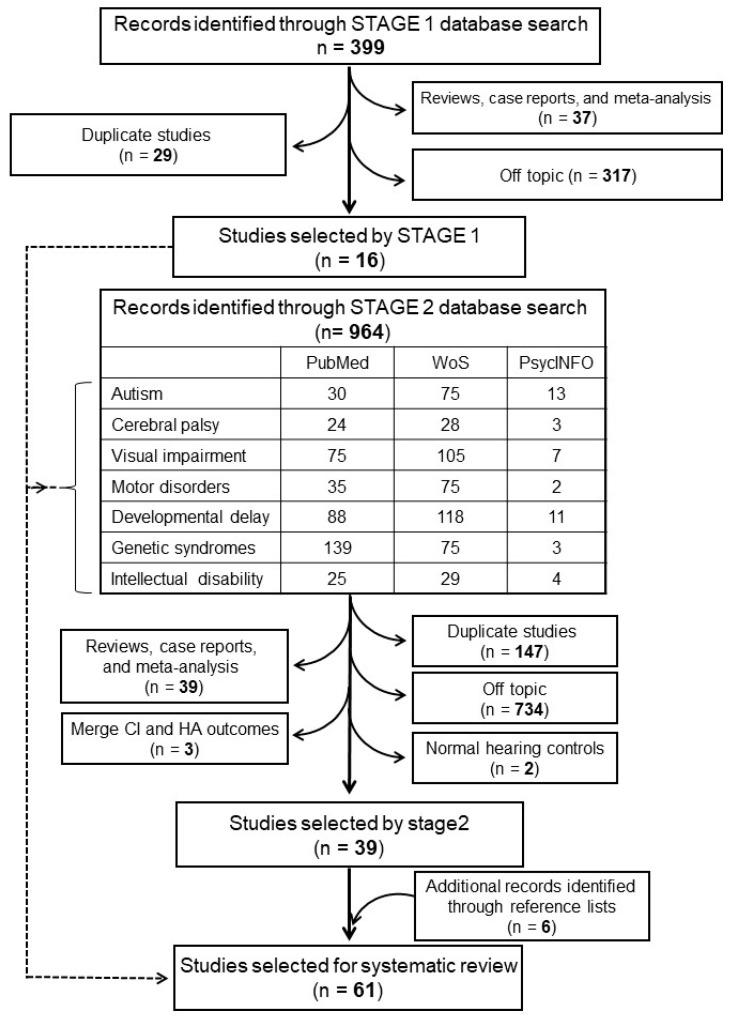
Flow chart of the systematic review process. CI, cochlear implant; HA, hearing aid.

A flow chart of our literature search is depicted in Figure 1. Stage 1 identified 16 articles listed in Supplementary Reference List S1. Additional disabilities assessed in these articles included autism spectrum disorder (ASD), cerebral palsy, visual impairment, motor disorders, developmental delay, genetic syndromes, and intellectual disabilities. By searching each one of these disabilities separately in Stage 2, we identified 39 additional articles, listed in Supplementary Reference List S2. Finally, by searching reference lists from reviews, we identified another 6 articles, listed in Supplementary Reference List S3, yielding a total of 61 articles. Among the selected articles, 13 studies including 271 cases and 900 controls fell into class 1 (Table 2); 19 studies with 496 cases and 1093 controls into class 2 (Table 3); 16 studies including 259 cases into class 3 (Table 4); and 13 studies with 191 cases into class 4 (Table 5). Table 2, Table 3, Table 4 and Table 5 summarize the relevant information for all of these studies, including the type of associated disability; the sample size of cases and controls; mean age at cochlear implantation (CI); duration of follow-up; measures used for auditory perception, language assessment, cognitive/adaptive skills, and quality of life; pre-CI differences between cases and controls; and post-CI outcomes.

Collectively, the most frequent disability associated with hearing loss in the 61 selected studies was “genetic syndromes/chromosomal abnormalities” (33/61, 54.1%); followed by “autism” (i.e., Autism Spectrum Disorder or ASD) in 26/61 (42.6%) studies; “developmental delay” (either motor or global) in 24/61 (39.3%) studies; “cerebral palsy” (22/61, 36.1%), “visual impairment” (12/61, 19.7%), and “intellectual disability”, assessed in 10/61 (16.4%) studies also including two reports addressing “learning disabilities” [33,44]; and finally “motor disorders” (6/61, 9.8%). These percentages refer to the number of articles in which each type of disability was clearly reported, as presented in Table 2, Table 3, Table 4 and Table 5 and do not take into account the frequent co-occurrence of multiple disabilities in a single case. Some studies listed in Table 2, Table 3, Table 4 and Table 5 included as “additional disabilities” other disorders that we decided to exclude from our stage 2 search (see Materials and Methods), namely, “ADHD” (8/61, 13.1%), “epilepsy”, (4/61, 6.6%), “behavioral problems”, and “medical disorders” (1/61, 1.6% each). Approximately half of the studies were focused on a single additional disability (31/61, 50.8%), whereas the remaining studies (30/61, 49.2%) assessed different additional disabilities by combining all cases together, or, in a few instances, analyzing them separately by condition, depending on sample size and experimental design.

From a methodological standpoint, the majority of studies (42/61, 68.9%) recruited samples smaller than 30 cases. Very small sample sizes (N < 10 cases) were especially frequent in class 3 and 4 studies (Table 4 and Table 5), whose results should be viewed with special caution and/or within the context of rare genetic disorders. The duration of follow-up was very variable: prospective follow-ups generally lasted 12–36 months, whereas observational studies assessed cases and controls who had received a CI up to more than 10 years prior. A variety of assessment tools was used in different studies to evaluate communication skills and speech production, auditory skills, cognitive level, adaptive skills, developmental trajectories, and quality of life (Table 2, Table 3, Table 4 and Table 5). A complete list of assessment tools and their primary reference is provided in Supplementary Table S1.

Almost all Class 1 and 2 articles provide consistent evidence that children with additional disabilities do benefit from CI. However, they display great interindividual variability in the extent of improvement and timing of response (Table 2 and Table 3). Furthermore, children with additional disabilities collectively improved to a lesser extent compared to hearing-impaired children without additional disabilities in at least 27/32 (84.4%) class 1 and 2 studies, and generally took a longer time to respond (Table 2 and Table 3). In reference to the degree of response in the auditory and language domains, three levels of beneficial effects can be derived from class 1 and 2 studies: (A) the vast majority of children (approximately 80–90%) achieved greater environmental sound awareness, leading to improved adaptive skills; (B) approximately half of the children appeared to improve in speech awareness and in receptive language; (C) a smaller minority developed some form of expressive language, which occasionally became the primary communication mode, but more often improved communication by conjoining sign language or other forms of augmentative alternative communication (Table 2 and Table 3). While these response rates are clearly below the achievements of children without additional disabilities, it is particularly noticeable that even children who do not develop expressive language usually improve in adaptive behaviors [16] and/or in environmental sound awareness [22,24]. In reference to the timing of the responses, children with additional disabilities often displayed a slower rate of improvement, possibly reaching the maximum response level 2–3 years after CI, as compared to control children reaching higher response levels at 12 months [16,29,37]. Evidence from class 3 and 4 articles, despite lower reliability or a more targeted focus, also converges upon these same conclusions, both in reference to a lower, yet sizable improvement in the auditory and language domains as compared to DHH children without additional disabilities and to a slower rate of response to CI (Table 4 and Table 5).

Additional converging evidence of the benefits of CI in children with additional disabilities, even in many of those who do not develop expressive language, comes from 11 studies assessing quality of life (QoL) after CI (Table 2, Table 3, Table 4 and Table 5), which was significantly improved in over 90% of all families. QoL represents a very relevant, though indirect, measure of treatment efficacy. Parents generally expressed satisfaction with the outcome of the implantation procedure, observing greater awareness of environmental sounds, propensity to communicate, social motivation, and interactions in their children [46,49,54,66]. Despite these positive results, the QoL of children with additional disabilities remains lower than the QoL of children without additional disabilities, especially in the domains of self-esteem, relationships with peers, and school [30,40,43,45].

Some studies suggest that, predictably, children with learning disabilities or ADHD may have greater rates of improvement and better outcomes after CI compared to children with ASD or cerebral palsy [7,17,23]. Caution must be exercised in attempting to move from “additional disabilities” to single disorders. In fact, studies are so heterogeneous that it is not possible to reach firm conclusions at this time, and many studies analyze “additional disabilities” by lumping together small numbers of cases with a variety of disorders. Perhaps a clearer picture can be reached regarding children with specific genetic syndromes, although these can also largely vary in terms of the degree of cognitive deficits and neurological impairment, which appear to be directly related to the CI outcome [65]. Children with Usher syndrome or Waardenburg syndrome may benefit from CI as much as children without any additional disability, if cognition is relatively preserved and intellectual disability is mild [7,32,37,41,44,72]. Children with CHARGE syndrome may also improve, but, in line with the severity of this syndrome, environmental sound awareness seems to be the most consistent benefit [48,51,61,66], while spoken language can be obtained when intellectual disability and neurological deficits are mild [51,55,69]. In addition, some results suggest that children with developmental delays receiving a CI, beyond auditory skills and communication modes, may also benefit in terms of their gross motor and social functioning, whereas problem solving, communication, and fine motor domains respond to a lesser extent [27].

Moving from a categorical to a dimensional approach, three important conclusions can seemingly be reached regardless of diagnosis: (A) the severity of intellectual disability (cognitive impairment) or developmental delay is inversely proportional to the degree of improvement in auditory and language skills after CI [34,35,36,48,55,60,64,65,71]. In this regard, purely motor deficits may exert little or no influence [35]. (B) Within each category of additional disabilities and each genetic syndrome, there are bound to be “responders” and “non-responders”, and this may depend on the severity of the additional disability, as documented in cerebral palsy [19,54,71], and in ASD, especially when associated with intellectual disability [23,70]. (C) Another diagnosis-independent factor that predictably influences outcome is age at the time of cochlear implantation [18,38,44,47,57,71]. This is comparable when children develop additional disabilities after CI, but tends to be delayed in children with early-onset disabilities, such as developmental delay, most cerebral palsies, and some genetic syndromes (Table 2, Table 3, Table 4 and Table 5) [18,57,68,69,71]. Early implantation in these conditions is associated with better outcomes and with greater rates of attainment of some degree of spoken language, to be associated with sign language as the primary mode of communication [38,48,71].

## 4. Discussion

An increasing number of children with different neurodevelopmental disorders are being treated with CI to provide access to sound in the presence of severe-to-profound hearing loss. In some cases, neurodevelopmental signs are already recorded at birth or during neonatal life, while in other cases, CI is performed before the overt clinical onset of behavioral and neurological symptoms. Consequently, an increasing number of studies are exploring the outcome of children with additional disabilities following CI.

The primary question that the studies reviewed herein aimed to answer is whether and to what extent it is justified to implant deaf children who also have other disabilities beyond hearing impairment. This literature is mainly audiological and not neuropsychiatric; hence, its focus is on hearing impairment, not on single neurodevelopmental disorders. Providing a systematic overview of the current literature has thus been challenging in at least two ways: (a) neurodevelopmental disorders are clinically different from each other, if assessed by categorically applying DSM-5 criteria, but in real life, they frequently co-occur in the same individual [74]. Furthermore, genetic syndromes associated with deafness often encompass ASD, intellectual disabilities, developmental delays, motor disorders, epilepsy, learning disabilities, and ADHD. Finally, these same disabilities are frequently clinical correlates of perinatal brain damage, which is not always assessed by MRI and can also yield different forms of cerebral palsy. Our stage 2 search strategy thus represents a necessary oversimplification of a very complex neurodevelopmental conundrum. Results should be interpreted while keeping this complexity in mind. (b) The definition of “outcome” is bound to vary significantly depending on which functions are assessed as dependent variables. Environmental sound awareness, verbal and non-verbal communication, expressive and receptive language, intellectual level, adaptive skills, social motivation and cognition, executive functions, motor skills, and sensory processing are bound to have different thresholds of liability to improvement following CI. Tests used to measure these functions are indeed not equivalent when it comes to their sensitivity to change over time in pre-/post-treatment studies, as has previously been analyzed in intervention studies of genetic syndromes like fragile X ([75] see table 2). Finally, they may be not suitable for assessing DHH children with associated disabilities, as the assessment of these children is particularly complicated due to the interaction between hearing loss, cognitive skills, and language development [6].

Aiming to overcome these hurdles, we have reviewed the literature by applying two distinct levels of analysis: first, “additional disabilities” were viewed as a single entity; then, we attempted to begin dissecting different trajectories, as described in the current literature for different disabilities. On the one hand, a unitary view of “additional disabilities” was encouraged not merely by methodological considerations, but also by converging genetic, neurobiological, teratogenetic, and clinical evidence of shared pathogenetic underpinnings among different neurodevelopmental disorders [76,77]. On the other hand, articles addressing the outcome trajectories of distinct additional disabilities have indeed provided initial, though still limited, evidence enabling us to detect at least some interesting trends.

Looking at additional disabilities as a collective entity, children with additional disabilities can be confidently expected to display greater interindividual variability in response to CI compared to children without additional disabilities. On average, they improve less and require a longer time to display clinically meaningful changes, but the range of response can vary widely (Table 2, Table 3, Table 4 and Table 5). The severity of cognitive disability/developmental delay appears especially influential on CI outcome [34,35,36,48,55,60,64,65,71]. Indeed, the presence of additional disabilities was found to represent the strongest significant independent factor affecting cognitive, language, and motor outcomes in DHH children with early auditory intervention [78]. In many regards, this result is not surprising: receptive and expressive language acquisition should be expected to proceed in parallel with the child’s developmental quotient and not with his or her chronological age. If the acquisition of other functions is limited and developmental stages are delayed, auditory and language skills should be expected to proceed at a similar pace. Moreover, “additional disabilities” are different forms of neurodevelopmental disorders, which are known to stem from abnormal cell migration, neuronal differentiation, neurite outgrowth, synaptic connectivity, and/or myelination [79]. These neurodevelopmental derangements, of genetic and/or epigenetic origin, affect not only multimodal sensory integration, sensory–motor coordination, executive functions, social cognition, and cognitive processing, but also brain plasticity, learning, memory, and the acquisition and maintenance of new skills [80,81]. Synaptopathies, for example, profoundly affect the capacity of neural circuits to undergo plastic changes, as is required for children to respond to any type of intervention [82]. For example, the dichotomy “responders” vs. “non-responders” has also been observed in children with ASD who are exposed to different forms of behavioral interventions [83] and may have significant genetic determinants, at least in some cases [84]. This clearly indicates that the neuroplastic underpinnings of successful exposure to novel sensory or environmental stimulation, including cochlear implantation, play a pivotal role in the clinical response to treatment and that these underpinnings are highly personalized.

Rather than focusing outcome assessments on traditional measures, such as verbal language development and cognitive skills, a more productive and realistic approach for children with additional disabilities would be to measure CI success primarily based on improved adaptive skills and quality of life. Instruments for testing auditory performance and speech intelligibility are designed for children without additional disabilities, and such outcomes do not seem to reflect the benefit in real life [11,16,30,46,54,62]. In fact, increased environmental sound awareness represented the most frequent positive outcome in the studies we have analyzed (Table 2, Table 3, Table 4 and Table 5). Enhanced access to environmental sounds is seemingly able to boost adaptive skills, as well as social and object interactions, in many children [11,16,30,46,54,62]. On these premises, some authors have suggested that the benefits of CI are best captured by parent QoL outcome instruments, in addition to standard measures [9], and in recent years studies the use of QoL questionnaires is increasing. In these studies, the implant has a positive influence on quality of life, despite the fact that for many children, spoken language remains an unrealistic target [46,49,54,66]. Most families observed a greater propensity to communicate in their children and an improvement in interaction, with greater awareness of environmental sounds [46,49,54,66]. For children with special needs, the aim of CI may not be to achieve open-set speech perception understanding, but rather to allow the child to feel part of their auditory environment. Nonetheless, the emotional burden on DHH children with additional disabilities remains sizable, and is not completely overcome by cochlear implantation [30,40,43,45]. These limitations should be considered when designing programs able to provide psychological support, especially in school settings and for peer relationship management.

The limited data contrasting specific disabilities suggests that, in general, children with learning disabilities or ADHD may have greater rates of improvement and better outcomes after CI compared to children with ASD or cerebral palsy [7,17,23], especially in the presence of severe cognitive disability and/or developmental delay [34,35,36,48,55,60,64,65,71]. Again, this general framework is not surprising, considering the neurobiological and genetic underpinnings of these conditions. However, some specificities should be carefully considered. One aspect which has seemingly not received attention to date is the relevance of sensory issues in ASD [85], including, but not limited to, auditory processing [86]. Sensory issues and their relevance in the quality of life of individuals with autism have only recently been fully recognized. Viewing these issues in terms of “hyper”- or “hypo”- sensitivity is an oversimplification, as the main overarching difficulty appears to be sensory integration [85,86]. Specifically for hearing, the spatial integration of sounds coming from multiple different sources and the temporal integration of intermittent sounds appear especially problematic in many autistic individuals [86]. Sensory issues should be assessed and analyzed pre- and post-CI, at a minimum using questionnaires such as the Short Sensory Profile [87] or similar questionnaires [88], in reference to all sensory organs of the individual. In fact, following CI, the preferential sensory channels used to explore the environment may change, as well as the intensity and characteristics of self-stimulation and stereotypic behaviors; all of this information could provide additional parameters signaling a successful implantation. Finally, the correlation between daily device use and auditory/language skills is strong in children without additional disabilities, but is less clear and more variable in cases with ASD [31]. Some children with ASD may inactivate their hearing devices more frequently than controls in order to better focus their attention on specific visual targets. In this case, this behavior would unveil not so much a reduced sensory threshold, but rather a defective sensory multimodal integration capacity [86]. Conceivably, using appropriate electrophysiological approaches, it may be interesting to assess pre-CI levels of connectivity between auditory and other neocortical/subcortical regions of interest in an attempt to predict which children will limit the use of their hearing devices to the point of making them useless.

Among other additional disabilities, cerebral palsy has been the object of several studies. Variable degrees of hearing impairment are not unusual in CP, with prevalence estimates ranging from 7 to 39% [89,90]. Forms of mild and moderate severity [19,60], especially athetoid [54], have been associated with better outcomes, whereas the presence of more severe cognitive impairment appears to be detrimental [29,34,71]. Finally, several studies either include some patients with genetic syndromes or focus on a single genetic syndrome among those more frequently associated with hearing impairment (Waardenburg syndrome, Usher syndrome, CHARGE syndrome, Bartter syndrome, Down syndrome). Collectively, even within single syndromes, there is great interindividual variability. Importantly, the definition of “responder” and “non-responder” may again depend on the degree of cognitive impairment and on the function measured to define the outcome (response in expressive language skills is typically less frequent as compared to improved adaptive behaviors) (Table 2, Table 3, Table 4 and Table 5). However, it is important to underscore that the current level of evidence, in our opinion, is not sufficient to reliably meta-analyze existing data in reference to single disabilities. In particular, the significant between-study heterogeneity in the age range at CI, sex distribution, clinical severity of additional disabilities, and outcome measures does not allow for the reliable pooling different data sets together to perform a meta-analysis. Until this approach can be applied, caution must be exercised in describing the outcomes of different additional disabilities in comparison to each another. The interesting preliminary trends we have outlined for some neurodevelopmental disorders and genetic syndromes indeed represent working hypotheses for future research more than firm conclusions at this time.

The literature investigating the influence of additional disabilities on the outcome of cochlear implantation has several limitations, which are reflected in the present work and must be distinguished from the limitations of our methodology. First, only a minority of published studies (13/61 = 21.3%) contains both pre- and post-implantation evaluations for both cases with additional disabilities and controls without additional disabilities (“class 1”). Secondly, studies largely differ in age range at CI; sample size; sex distribution; clinical severity of additional disabilities; and duration of the follow-up period, which ranges from 1 to 3 years (Table 2). Also, very different assessment measures have been used for auditory perception, language and communication skills, cognitive level, quality of life, motor function, and/or global development in different studies (Table 2, Table 3, Table 4 and Table 5). Thirdly, neurodevelopmental disorders tend to co-occur, and information on single-patient co-morbidity patterns is completely lacking. Fourthly, no study has recruited children with normal hearing and additional disabilities as a control group so as to better compare the benefit of the CI on behavior and quality of life, rather than the negative impact of additional disabilities on CI outcome. Finally, cases belong to a variety of “additional disabilities” in the vast majority of studies (Table 2, Table 3, Table 4 and Table 5); combining patients with different neurodevelopmental disorders and genetic conditions into a single sample makes it impossible to reach firm conclusions on any single neurodevelopmental disorder, and forces us, at this stage, to look collectively at “additional disabilities” as a whole. These limitations are intrinsic to the currently available literature and are inevitably reflected in this systematic review. On the other hand, one limitation of the present work is that it is designed as a comprehensive overview of the field and a descriptive summary of major trends, not as a quantitative metanalysis by a single function (hearing results, language comprehension, listening skills, etc.) or additional disability. Another possible limitation specific to our approach may consist of the exclusion of “ADHD”, “epilepsy”, “behavioral problems”, and “medical disorders” from the Stage 2 search terms. Our reasons for making this choice are explained in Section 2.1. Nonetheless, we cannot exclude that some additional articles containing useful information could have been collected, also using these terms in Stage 2 to define single additional disabilities. Despite these limitations, our systematic review has at least two strengths which also deserve to be acknowledged. One strength lies in the two-stage approach we used for our literature search. Performing a Stage 2 search by empirically deriving search terms from the articles selected in stage 1 proved to be a solid and reliable strategy which provided a consistent number of articles, allowing us to gather a critical mass of useful information. Another strength lies in the distinction of four levels of evidence based on the experimental design of the selected articles, distinguished accordingly into classes 1–4 (Table 1). This allows readers to better “weigh” the evidence provided by each study listed in Table 2, Table 3, Table 4 and Table 5.

## 5. Conclusions

This systematic review provides consistent evidence that, collectively, children with additional disabilities do benefit from CI, and that they should by no means be excluded from a cochlear implantation program. On the one hand, using traditional assessment tools of auditory and language function as outcome measures, children with additional disabilities predictably improve to a lesser extent and at a slower pace compared to children without additional disabilities. However, enhanced environmental sound awareness, which was reported in the vast majority of studies, translates into increased adaptive skills in different contexts, enhanced social motivation and intent to communicate, and better quality of life for the child and for family members. This does not exclude at all that some valuable receptive and expressive language gains may be obtained, but requires a change in paradigm both among the clinicians who prescribe CI procedures and in family members, which need accurate counseling in order to ensure realistic expectations. The QoL data summarized in this review are especially positive in terms of witnessing how family members may be much more sensitive than auditory and language tests to the improvement produced by CI in their children with deafness and additional disabilities. Hence, despite some limitations, our systematic review reached a reliable conclusion in favor of the usefulness of CI in hearing-impaired children with additional disabilities, and hopefully provides information which can be of use to clinicians. It also spurs interest in follow-up studies aimed at meta-analytically integrating available data for specific functions, such as communication skills and adaptive functioning, or for single neurodevelopmental disorders and genetic syndromes, once sufficient data become available.

## Figures and Tables

**Table 1:** Study classification into four classes, based on experimental design and strength of evidence.

Study Classification	Cases	Controls	Pre-CI Data	Post-CI Data
Class 1	Yes	Yes	Yes	Yes
Class 2	Yes	Yes	No	Yes
Class 3	Yes	No	Yes	Yes
Class 4	Yes	No	No	Yes

**Table 2:** List of “class 1” articles selected after both stage 1 and stage 2, with pre- and post-cochlear implantation (CI) characteristics of children with or without additional disabilities (“cases” and “controls”, respectively).

Ref.	Additional Disability	Cases and Controls		Follow-Up in Years (y)	Assessment Measures	Pre-Implant	Post-Implant
		N	Mean Age at CI (Range) in Years (y)				
[15]	Motor dev. delay	28 cases 234 contr	3.54 y 4.22 y	2 y	Auditory skills: CAP, Language and speech skills: SIR	Cases = controls	Equal improvement in auditory and language skills in cases and controls.
[16]	ASD Cerebral palsy Dev. delay Motor impairment Genetic syndromes	23 cases 23 contr	2 y (9 m–4 y) 1.8 y (10 m–3.3 y)	2 y	Auditory skills: IT-MAIS Language and speech skills: PLS-4, communication mode Cognitive functioning: DAYC/Bayley Behavior: VABS	Cases = controls, except for cognitive level	Improved scores in both cases and controls: cases < controls at 12 months and cases = controls at 24 months. Cases improved more in receptive than in expressive language. Cases who did not improve in language, still improved in adaptive behaviors.
[17]	ADHD ASD Cerebral palsy Learning disability	31 cases 157 contr	2.5 y 2.3 y	3 y	Oral language (expressive and receptive): RDLS, communication mode. Behavior: CBCL	Language: cases < controls, except for ADHD cases = controls; Externalizing behaviors: cases = controls, except for CP > controls Internalizing behaviors: cases = controls	Improved language in both cases and controls, but cases made slower progress, especially in the presence of ASD. At 36 months: ASD < cerebral palsy < ADHD = learning disability = controls. Externalizing behaviors: cases > controls Internalizing behavior: cases = controls
[18]	Dev. delay	12 cases 24 contr	2.1 y 1.33 y	2.7 y 1.8 y Normalized at 2 y	Language and speech skills: PLS Learning skills: MSEL Behavior: VABS QoL: PSI	Cases = controls	Cases < controls, with partial improvement only in fine motor domain (MSEL). Later age at implantation negatively influenced intelligence and behaviour, contributing to worse outcomes in cases compared to controls.
[19]	Cerebral palsy	8 cases 8 contr	1.8 y (1.33–2.4 y) 1.7 y (1.1–2.33 y)	2 y	Auditory skills: CAP, Ling’s sounds test Language and speech skills: SELSI Cognitive functioning: VSMS/Bayley Motor functioning: GMFCS	Cases = controls	- Mild-to-moderate CP: Four cases improved almost as much as controls in auditory and language skills. - Severe CP: Four cases remained well below controls, but improved in awareness of environmental sounds.
[20]	Dev. delay ASD Genetic syndromes Cerebral palsy Visual impairment	19 cases 230 contr	3.6 y (1.4–10.5 y) 4.1 y (6 m–15.9 y)	1 y	Auditory skills: CAP, MAIS, LiP Language and speech skills: MUSS	Cases < controls	Improved scores in both cases and controls, but cases < controls
[21]	Intellectual disability ADHD	14 cases 14 contr	5.8 y (1.8–11.1 y) 6.1 y (1.0–10.0 y)	1 y	Auditory skills: CAP, K-Ling	Cases < controls	Improved scores in both cases and controls, but cases < controls
[22]	ASD	15 cases 15 contr	3.0 y (1.5–5.5 y) 3.5 y (1.5–15 y)	8.3 y cases 8.2 y contr (yrs with CI)	Auditory skills: CAP, ESP, MLNT Language and speech skills: PBK, communication mode.	Cases = controls	Improved scores in both cases and controls, but cases < controls. Parents reported greater awareness of the child’s environment.
[23]	Intellectual disability ASD with intellectual disability	8 cases 5 contr	2.1 y (1.5–3.7 y) (cases and controls together)	2 y	Auditory and communication skills: Enjoji Scale of Infant Analytical Development Cognitive: Tanaka-Binet test.	Cases < controls	Improved scores in controls only. Cases with ID and especially with ASD and intellectual disability displayed increasing delays for all measured functions.
[24]	ASD Visual impairment Motor disorder Dev. delay Genetic syndromes Intellectual disability	38 cases 26 contr	4.7 y 4.1 y	2 y	Auditory skills: MAIS, LIP Language and speech skills: MUSS Cognitive: LIPS-R, GMDS	cases < controls at MAIS cases = controls	Improved scores in both cases and controls, depending on disability type and severity. Children who did not develop language, improved in non-verbal cognitive skills, displaying greater awareness of their environments.
[25]	ASD Dev. delay Genetic syndromes Cerebral palsy Epilepsy	40 cases 40 contr	3.7 y (0.9–12.0 y) 3.6 y (1.0–7.0 y)	1 y	Auditory skills: CAP, MAIS Language and speech skills: SIR, MUSS	cases = controls	Improved scores in both cases and controls, but cases < controls
[26]	Dev. delay	32 cases 99 contr	1.1–2.1 y 1.1–1.7 y	2 y	Auditory skills: LEAQ Language and speech skills: PLS Learning skills: MSEL Behaviour. VABS QoL: PSI	cases = controls	Improved scores in both cases and controls, but cases < controls.
[27]	Dev. delay	26 cases 25 contr	1.0 y (1.0–1.25 y) 1.0 y (1.0–1.25 y)	1 y	Communication level, motor delay: ASQ-3	Cases < controls for all subtests	Cases = controls for gross motor and social development Cases < controls for communication, fine motor, problem solving.

List of abbreviations: ADHD: Attention Deficit/Hyperactivity Disorder; ASD: Autism Spectrum Disorder; ASQ-3: Ages and Stages Questionnaire third edition; Bayley: Bayley Scales of Infant and Toddler Development; CAP: Categories of Auditory Perception; CBCL: Child Behavior Checklist; DAYC: Developmental Assessment of Young Children; Dev.: developmental, ESP: early speech perception test; GASP: Glendonald auditory screening procedure; GMDS: Griffith Mental Developmental Scale; GMFCS: Gross Motor Function Classification System; HINT: hearing in noise test; LEAQ: LittlEARS Auditory Questionnaire; LiP: listening progress score; LIPS-R: Leiter International Performance Scale-Revised; MAIS: meaningful auditory information scale; MLNT: Multisyllabic Lexical Neighborhood Test; MSEL: The Mullen Scales of Early Learning; MUSS: Meaningful use of speech scale; PBK: Phonetically Balanced Kindergarten test; PLS: Preschool Language Scale; PSI: Parental Stress Index; RDLS: Reynell Developmental Language Scales; SELSI: Sequenced Language Scale for Infants; SIR: Speech Intelligibility Rating; VABS: Vineland Adaptative Behaviour Scale Ed II; VSMS: Vineland Social Maturity Scale.

**Table 3:** List of “class 2” articles, with only post-cochlear implantation (CI) characteristics of children with or without additional disabilities (“cases” and “controls”, respectively).

Ref.	Additional Disability	Cases and Controls		Follow-Up in Years (y)	Assessment Measures	Post-Implant
		N	Mean Age at CI (Range) in Years (y)			
[7]	Dev. delay ASD ADHD Usher syndrome	25 cases 25 contr	3.65 y (0.4–10.9 y) 3.42 y	1.0–8.3 y	Auditory skills: MAIS Language and speech skills: MUSS, communication mode (pre-CI data provided only for communication mode).	Improved scores in both cases and controls, but cases improved less than controls. Post-CI oral communication mode acquired by 10 (40%) cases and 24 (96%) controls. ASD < ADHD = Dev. delay < Usher syndrome = controls.
[28]	ADHD Cerebral palsy (mild) Epilepsy	15 cases 16 contr	3.32 y (cases and contr together)	Mean 4.2 y	Auditory skills: Spondee test (two-syllable word) presented aurally, visually, or both	Controls > cases with one additional disability > cases with two or more additional disabilities. Note: severe motor or mental disorder was a cause for exclusion
[29]	ASD Intellectual disability Dev. delay Cerebral palsy	17 cases 29 contr	1.6 y (0.9–2.0 y) 1.4 y (0.8–2.0 y)	2.0 y	Auditory and language skills: Little-EARS	Post-CI scores improved over time in both cases and controls. Non-verbal auditory receptive behavior—cases < controls at 3–9 m, cases = controls at 24 m. Semantic auditory behavior—cases < controls at all time points (3–24 m). Expressive language—cases < controls at all time points, but cerebral palsy much slower and less responsive than other groups.
[30]	ASD Dev. delay Cerebral palsy	43 cases 49 contr	2.25 y (0.65–5.4 y) 2.0 y (0.55–5.6 y)	3.0 y 3.5 y	Language and speech skills: communication mode QoL: Kiddy KINDL^R^	Communication mode: improved scores, although cases < controlsQuality of life: - Cases < controls in self-esteem, friends, and school domains. - Cases = controls at physical, emotional, and family domains.
[31]	ASD Genetic syndromes Dev. delay Cerebral palsy ADHD	16 cases 49 contr	2.2 y (1–4.2.0 y) 1.9 y (0.6–4.3 y)	1.6 y (0.6–3.4 y) 1.6 y (0.1–3.8 y)	Auditory skills: ASC Language and speech skills: SRIQ, Receptive–Expressive Emergent Language Test III Ed., Oral and Written Language Scales II Ed, communication mode	Improved scores in both cases and controls, but cases < controls. Correlation between daily device use and auditory/language skills is strong in controls, but less clear and more variable in cases.
[32]	Waardenburg syndrome (including six cases with ASD, dev. delay or dyspraxia, visual impairment, or learning disabilities).	14 cases 48 contr	1.6 y 1.3 y	8.3 y	Language and speech skills: RDLS LQ, Phoneme score (retrospective study)	No significant difference between 14 cases with Waardenburg Syndrome (WS) and 48 controls, in the absence of additional disabilities. Variability in the WS sample was due to 6/14 (42.3%) cases with additional disabilities. The presence of additional disabilities exerted a significant negative effect on expressive and receptive language outcomes.
[33]	ASD Intellectual disability Cerebral palsy Visual impairment Motor impairment	16 cases 61 contr	4.0 y 4.5 y	5.0 y	Auditory Skills: CAP, ASC, SRIQ Language and Speech skills: Receptive–Expressive Emergent Language Test, III Ed., Oral and Written Language Scales, II Ed.	Improved scores in both cases and controls, but cases < controls for receptive and expressive language. Cases < controls in school placement: Cases: 1 (6.3%) in regular classes, 11 (68.8%) in special classes, 4 (25.0%) in special education Contr: 42 (68.8%) in regular classes, 17 (27.9%) in special classes, 2 (3.3%) in special education
[34]	Dev. delay Cerebral palsy Visual impairment Genetic syndromes ASD	70 cases 22 contr	3.2 y (0.9–9.8 y) 4.5 y (0.4–12.5 y)	6.4 y 7.3 y	Auditory skills: Alternative Scale (parents observation), LittleEARS, Ling’s sounds Language and speech skills: ESP, MSEL, TROG	Improved scores in both cases and controls, but cases < controls in the presence of cognitive deficits: 48 cases with cognitive dysfunction < 22 cases with only physical dysfunction = 22 controls with auditory neuropathy spectrum disorder.
[35]	Motor disorders Behavioral problems Learning disorders	64 cases 224 contr	2.2 y (cases and controls together)	6.4 y	Language and speech skills: RDLS, SELT, mode of communication	Improved scores in both cases and controls, but with different outcomes: - Receptive language: cases with learning disability < motor disorders = controls - Expressive language, words: all cases < controls-Expressive language, sentences: learning disability < motor disorders = controls
[36]	Dev. delay Cerebral palsy Visual impairment ASD ADHD Genetic syndromes	29 cases 57 contr	0.0–16.0 y	1.0 y	CAP	Improved scores in both cases and controls, but cases < controls, especially in the presence of developmental delay.
[37]	Genetic Syndromes (Waardenburg s. cases and Cx26/30 mutation controls).	30 cases 85 contr	4.8 y (1.3–16.0 y) 4.7 y (1.3–16.5 y)	4.0 y	Language and Speech skills: CSW-OSW, PPVT Cognitive: Weschler Intelligence Scale, Weschler Preschool and Primary Scale of Intelligence-III, Kaufman Assessment Battery for Children	Improved scores in both cases and controls. Perceptive and linguistic evaluations for both populations were of good quality, but lexical evaluation showed residual language difficulties in both groups. Cases < controls at 12 m, except for lexical comprehension (cases > controls). Cases = controls at 24 m in CSW-OSW, cases < controls in comprehension. Cases = controls at 36 m Cases = controls at 48 m.
[38]	Genetic syndromes (Down syndrome)	9 cases 220 contr	6.3 y (1.75–10.6 y) 4.3 y (0.75–13.9 y)	14.9 y (13.1–18.3 y)	Auditory skills: CAP, MUSS Language and speech skills: MUSS, SIR	Improved speech and receptive language in both cases and controls, but cases < controls. Earlier implantation benefited both cases and controls.
[39]	ASD	4 cases 10 contr	1.3 y 1.35 y	5.0 y	Auditory skills: CAP Language and speech skills: SIR	Improved scores in both cases and controls, but cases much less than controls.
[40]	Dev. delay	17 cases 35 contr	3.7 y 3.5 y	5.5 y 4.9 y	QoL: HRQoL, PVEIQ questionnaire	Improved scores in both cases and controls, but cases < controls in all QoL domains.
[41]	Genetic syndromes Visual impairment	13 cases 12 contr	5.25 y (cases and controls together)	3.0 y	Auditory skills: CAP Language and speech skills: SIR, communication mode	Equal improvement in all syndromic cases (Waardenburg s., Usher s., Dandy–Walker s. and albinism) compared to non-syndromic controls.
[42]	ADHD	19 cases 23 contr	2.1 y (0.33–5.0 y)	5.0 y	Auditory skills: NDS Language and speech skills: NDS QoL: ASQ	Improved scores in both cases and controls, but cases < controls for all expressive and receptive language measures, and for all developmental domains except for gross movement (cases = controls).
[43]	Genetic syndromes (Usher syndrome)	23 cases 30 contr	2.9 y (0.9–4.7 y) 4.1 y (1.8–6.0 y)	1.0 y	Auditory skills: CAP Language and speech skills: SIR QoL: HR-QOL	Improved scores in both cases and controls, but cases < controls for auditory skills, speech, and hearing-related quality of life.
[44]	Genetic syndromes (Usher syndrome)	35 cases 46 contr	6.3 y (0.3–17.6 y) (cases and controls together)	10 y	Auditory, speech, and language skills: CAP, SIR, MAIS, MUSS	Equal improvement in expressive and receptive language in both cases and controls. Greater improvement was recorded if cases and controls received CI at < 3 yrs old.
[45]	Visual impairment Motor impairment Dev. delay Cerebral palsy ASD	37 cases 52 contr	3.8 y (0.7–11.7 y) (cases and controls together)	7.4 y (2.0–14.3 y)	Language and speech skills: mode of communication QoL: PAQL	DHH children with additional disabilities achieved lower quality of life overall, particularly in “communication and independence”, “emotional well-being”, and “acceptance by peers”.

List of abbreviations: ADHD: Attention Deficit/Hyperactivity Disorder; ASC: Auditory skill Checklist; ASD: Autism Spectrum Disorder; ASQ: Ages and Stages Questionnaire; Bayley: Bayley Scales of Infant and Toddler Development; CAP: Categories of Auditory Perception; CBCL: Child Behavior Checklist; CSW-OSW: closed test, open set words; DAYC: Developmental Assessment of Young Children; DDST-II: The Denver Developmental Screening Test II; ESP: Early Speech Perception Test; GASP: Glendonald auditory screening procedure; GMDS: Griffith Mental Developmental Scale; GMFCS: Gross Motor Function Classification System; HINT: hearing in noise test; HROoL: health-related quality of life; LiP: listening progress score, MAIS: Meaningful Auditory Information Scale; MLNT: Multisyllabic Lexical Neighborhood Test; MSEL: The Mullen Scales of Early Learning; MUSS: Meaningful Use of Speech Scale; NDS: Newsha Developmental Scale; PKB: Phonetically Balanced Kindergarten test; PLS: Preschool Language Scale; PPVY: Peabody Picture Vocabulary Test; PSI: Parental Stress Index; RDLS: Reynell Developmental Language Scale; SELT: Schlichting Expressive Language Test; SELSI: Sequenced Language Scale for Infants; SIR: Speech Intelligibility Rating; SRIQ: Speech Recognition Index in Quiet; TROG: Test for Reception of Grammar; VABS: Vineland Adaptative Behaviour Scale Ed II; VSMS: Vineland Social Maturity Scale.

**Table 4:** List of “class 3” articles, including pre- and post-cochlear implantation (CI) characteristics of children with additional disabilities (“cases” only).

Ref.	Additional Disability	Cases		Follow-Up in Years (y)	Assessment Measures	Pre-Implant	Post-Implant
		N	Mean Age at CI (Range) in Years (y)				
[11]	Dev. delay Motor delay Cognitive impairment	6 cases	3.5 y (1.15–11.2 y)	1.0 y	Auditory skills: PEDI Language and speech skills: PEDI Cognitive: RGDS, Developmental Profile-3, NVCQ	No auditory or language skills	Improved functional and social abilities at 1 y. Improved receptive and expressive language at 1 y, with no change in language quotients.
[46]	Learning disabilities Motor dev. delay Cerebral palsy Genetic syndromes	16 cases	3.9 y (1.8–13.1 y)	3.0 y	Auditory skills: CAP Language and speech skills: SIR QoL: GCBI	No auditory or language skills	No improvement (6 cases) or low achievement (10 cases) in auditory and language skills. Improved QoL, especially in the learning and vitality domains.
[47]	ASD Intellectual disability Dev. delay Cerebral palsy Epilepsy Genetic syndromes Visual impairment	10 cases	4.2 y (2.0–13.1 y)	7.0 y	Auditory skills: CAP Language and speech skills: SIR, mode of communication	No auditory or language skills	The outcome was highly variable: 1/10 (10%) developed spoken language; 3/10 (30%) used some speech and some sign language; and 6/10 (60%) used non-verbal means of communication.
[48]	Genetic syndromes (CHARGE syndrome)	13 cases	1.1 y (0.9–1.4 y)	4.0 y (0.4–10 y)	Auditory skills: IT-MAIS, LittlEARS, GASP, CNC Language and speech skills: PLS-5, mode of communication	No auditory or language skills	Improved scores in 12/13 (92.3%) cases. Environmental sound awareness seemed to be the most consistent benefit. Early and bilateral CI may be more effective.
[49]	Intellectual disability ADHD/ADD Cerebral palsy Epilepsy	50 cases	4.1 y (1.8–13.3 y)	3.0 y	Auditory skills: P.CA.P., TIPI1, TIPI2, DADQ Language and speech skills: ESP, DADQ, GASS, mode of communication QoL: DADQ Behavior: CBC	No auditory or language skills	Improved scores in all auditory and speech domains, but mild intellectual disability improved more than moderate/severe. Parents’ satisfaction (DADQ): 48/50 (96%) would recommend a CI for a child with a similar impairment.
[50]	ASD Cerebral palsy Motor dev. delay Visual impairment Cognitive impairment	46 cases	0.8–6.2 y	≥5.0 y	Auditory skills: EarlyCaLL Language and speech perception: EarlyCaLL Behavior: EarlyCaLL	No auditory or language skills	Improved scores in all domains for cognitively impaired cases, not for children also with ASD. No case achieved independent management of the CI.
[51]	Genetic syndromes (CHARGE syndrome) Intellectual disability	7 cases	5.75 y (0.5–12 y)	3.3 y (1.3–6.0 y)	Auditory skills: ESP Language and speech perception: GASP, SIR, mode of communication	No auditory or language skills	Two cases developed spoken language; three had increased environmental sound awareness; two with progressive hearing loss maintained verbal language.
[52]	Genetic syndromes (CHARGE syndrome)	6 cases	4.9 y (2.0–9.8 y)	5.0 y	Auditory skills: MAIS, CAP Language and speech skills: SIR	No auditory or language skills	All patients improved in auditory and speech scores, except for one case
[53]	ASD Genetic syndromes	22 cases	2.8 m (0.8–12.0 y)	10.0 y	Auditory skills: CAP Language and speech skills: CL, mode of communication	No auditory or language skills	Auditory skills and receptive language: 19/22 (86.4%) improved; 3/22 (13.6%) showed no change. Expressive language skills: 4/22 (18.2%) improved; 18/22 (81.8%) had minimal or no improvement.
[54]	Cerebral palsy	9 cases	3.3 y (1.8–5.1 y)	1.58 y (1.1–2.4 y)	Auditory skills: GASP, MAIS Language and speech skills: Delgado Test, MUSS, mode of communication QoL: PEDI, GMFCS	No auditory or language skills	Variable outcome, generally better for auditory skills than for spoken language. Improved scores for functional skills and QoL. Children with athetoid cerebral palsy may develop better skills.
[55]	Genetic syndromes (CHARGE syndrome)	5 cases	3.1 y (1.5–4.5 y)	2.4 y (12–53)	Auditory skills: IT-MAIS Language and speech skills: ESP, mode of communication	No auditory or language skills	Improvement in auditory skills and receptive and expressive language, proportional to cognitive and neurological deficits.
[56]	ASD	30 cases	3.5 y (0.8–11.8 y)	10.5 y (1.4–21.6 y)	Auditory skills: MAIS, CNC Language and speech skills: ESP, PBK, mode of communication Behaviour: Social engagement, Parent survey	No auditory or language skills	Improved auditory scores. Improved receptive language in 15/30 (50%) cases. Communication only by spoken language in 9/30 (30%) and by sign and spoken language in 4/30 (13.3%). Improved social engagement in 25/30 (83.3%).
[57]	Genetic syndromes (Usher syndrome)	6 cases (excl. 4 adults)	11.7 y (5.0–16.0 y)	1.0 y	Language and speech skills: ESP, mode of communication	No auditory or language skills	Modest improvements in auditory and speech perception skills due to late implantation.
[58]	Genetic syndromes (Waardenburg syndrome)	5 cases	1.0 y (0.7–1.75 y)	2.0 y	Auditory skills: IT-MAIS Language and speech skills: MUSS	No auditory or language skills	Improved auditory and language skills, although below same-age typically-developing children.
[59]	Genetic syndromes (CHARGE syndrome)	7 cases	-	1.0–3.0 y	Audiological skills: SRS Language and speech skills: Bates test, mode of communication	No auditory or language skills	Delayed and limited improvement.
[60]	Dev. delay Cerebral palsy Visual impairment Genetic syndromes	21 cases	4.3 y (1.6–11.7 y)	1.0 y	Auditory skills: IT-MAIS, GASP, ESP, HINT Language skills: PKB, mode of communication	No auditory or language skills	Mild/moderate dev. delay: significant improvement in auditory skills and in spoken language. Severe dev. delay: greater environmental sound awareness with large interindividual variability in improvement of auditory skills; little or no improvement in spoken language.

List of abbreviations: ADD: Attention Deficit Disorder; ADHD: Attention Deficit/Hyperactivity Disorder; ASD: Autism Spectrum Disorder; Bayley: Bayley Scales of Infant and Toddler Development; CAP: Categories of Auditory Performances; CBC: Child Behaviour Checklist; CL: Categories of Language; DADQ: Deafness and Additional Disabilities Questionnaire; DAYC: Developmental Assessment of Young Children; Early CALL: Nottingham early cognitive and listening links; ESP: early speech perception test; GASP: Glendonald auditory screening procedure; GASS: Grid Analysis of Spontaneous Speech; GCBI: Glasgow Children’s Benefit Inventory, GMDS: Griffith Mental Developmental Scale, GMFCS: Gross Motor Function Classification System, HINT: hearing in noise test, LiP: Listening progress score; MAIS: Meaningful Auditory Information Scale; MLNT: Multisyllabic Lexical Neighborhood Test; MSEL: The Mullen Scales of Early Learning; MUSS: Meaningful Use of Speech Scale; PEDI: Pediatric Evaluation of Disability Inventory; PBK: Phonetically Balanced Kindergarten test; PLS: Preschool Language Scale; PLS-5: Preschool Language Scale-5; PSI: Parental Stress Index; RDLS: Reynell Developmental Language Scales; SELSI: Sequenced Language Scale for Infants; SIR: Speech Intelligibility Rating; SRS: Geers and Moog Speech Reception Score; VABS, Vineland Adaptive Behavior Scales; VSMS: Vineland Social Maturity Scale.

**Table 5:** List of “class 4” articles, including only post-cochlear implantation (CI) characteristics of children with additional disabilities (“cases” only).

Ref.	Additional Disability	Cases		Follow-Up in Years (y)	Assessment Measures	Post-Implant
		N	Mean Age at CI (Range) in Years (y)			
[61]	Genetic syndromes (CHARGE syndrome)	6 cases	3.1 y (1.3–5.3 y)	-	Auditory skills: CAP, Ling’s test, EARS (LiP, MTP6 and MTP12, BIS4 and BIS12, GASP), communication mode	Variable improvement, greater in auditory/perception skills than in spoken language.
[62]	ASD	6 cases	1.8 y (1.4–2.1 y)	5.9 y (3.6–8.5)	Auditory skills: Ling’s test Language and speech skills: Onomatopoeic word test, Parents Questionnaire, communication mode.	Delayed and limited improvement in receptive and expressive language. Reduced anxiety; parents reported benefits in family interactions.
[63]	Genetic syndromes (Down syndrome)	8 cases	2.6 y (0.9–5.3 y)	7.5 y (3.5–12)	Auditory skills: CAP Language and speech skills: SIR	General improvement, greater in auditory/perception skills than in spoken language.
[64]	ADHD ASD Cerebral palsy Dev. delay Genetic syndromes	19 cases	0.7 y	3.0 y	Auditory skills: IT-MAIS Language and speech skills: GASP, LNT, MLNT, PBK, IT-MAIS, mode of communication	Improvement in auditory and language skills commensurate with the severity of the additional disability. Cases with known disabilities at the time of CI may benefit less than cases who develop additional disabilities after CI.
[65]	Genetic syndromes	38 cases	3.3 y (1.25–18.0 y)	8.0 y (median) (≥1.5 y)	Auditory skills: SRS Language and speech: BKB, mode of communication	Improvement of auditory and language skills commensurate with the severity of cognitive impairment.
[66]	Genetic syndromes (CHARGE syndrome)	12 cases	3.5 y (1.7–8.2 y)	4.7 y (1.5–10.1)	Language and speech skills: ESP, LNT, MLNT, mode of communication QoL: Parents perceived benefits.	Auditory skills improved, although slowly: 10/12 (83%) children achieved environmental sound awareness and 6/12 (50%) achieved some speech perception. Benefits reported by the parents of 10/12 (83%) children.
[67]	Genetic syndromes (KID syndrome)	2 cases	5.0 y (3.0–8.0 y)	10.0 y	Auditory skills: perception tests Language and speech skills: IOWA, Matrix Level B closed set Sentence Test, SIR, Grammatical Analysis of Elicited Language, Pre-Sentence Level, Mode of communication.	One case developed fluent spoken language; the second case required explantation of the CI for wound infection and now uses sign language and lip reading.
[68]	Genetic syndromes (Infantile Bartter Syndrome)	5 cases	4.2 y (1.4–7.0 y)	8.9 y (6–12.5 y)	Auditory skills: CAP Language and speech skills: Freiburg Monosyllabic Word Test, Hochmair Schulz Moser sentence test in quiet.	Partial development of receptive language (some speech understanding in quiet) and expressive language, limited by the general health status (i.e., renal failure) and late referral for CI.
[69]	Genetic syndromes (CHARGE syndrome)	10 cases	5.6 y (0.7–16.0 y)	1.0–12.0 y	Auditory skills: CAP Language and speech skills: mode of communication.	Three children with pre-CI verbal language maintained verbal language post-CI; children without pre-CI verbal language developed signs and language in two cases, only signs and gestures in four cases, and instrumental use of others in one case.
[70]	ASD Genetic syndromes	22 cases	2.6 y (1.0–8.0 y)	2.0 y (0.2–7.1 y)	Auditory skills: Speech perception and expression categories. Language and speech skills: Speech perception and expression categories, mode of communication.	Some verbal communication in 13/22 (59.1%); only non-verbal communication (signs or PECS) in 6/22 (27.3%); no communication in 3/22 (13.6%) with very severe forms of ASD.
[71]	Cerebral palsy	5 cases	2.8 y (1.7–3.8 y)	2.3 y (0.1–3.2 y)	Auditory skills: IT-MAIS, classification of hearing. Language and speech skills: McArthur CDI, classification of language.	Improvement in auditory skills and speech in all cases, to a larger extent if operated on at an earlier age and with less or no cognitive impairment.
[72]	Genetic syndromes (Usher syndrome)	26 cases	3.3 y (0.5–11.6 y)	7.8 y (0.9–15.6)	Language and speech skills: ESP, MLNT, LNT, PBK, CNC, HINT, mode of communication	Positive outcome in the majority of cases: Open-set speech discrimination in 24/26 (92.3%) cases. Only or primarily oral communication mode in 18/26 (69.2%) cases.
[73]	Cerebral palsy Dev. delay Genetic syndromes Medical disorders	32 cases	2.0 y (0.6–4.75 y)	2.0 y	Auditory skills: CAP, Speech and language level: LittlEARS, ELFRA	Scores improved over time, more in receptive than in expressive language.

List of abbreviations: ADHD: Attention Deficit/Hyperactivity Disorder; ASD: Autism Spectrum Disorder; Bayley: Bayley Scales of Infant and Toddler Development; BKB: Bamford, Kowal and Bench speech reception score; CAP: Categories of Auditory Perception; CBC: Child Behavior Checklist; CL: Categories of Language; DADQ: Deafness and Additional Disabilities Questionnaire; DAYC: Developmental Assessment of Young Children; Early CALL: Nottingham early cognitive and listening links; ELFRA(Elternfragebogen ¨ für die Früherkennung von Risikokindern); ESP: Early Speech Perception; GASP: Glendonald auditory screening procedure; GASS: Grid Analysis of Spontaneous Speech; GCBI: Glasgow Children’s Benefit Inventory; GMDS: Griffith Mental Developmental Scale; GMFCS: Gross Motor Function Classification System; HINT: Hearing In Noise Test; KID: keratitis–ichthyosis–deafness syndrome; LiP: listening progress score; LNT: Lexical Neighborhood Test; MAIS: Meaningful Auditory Information Scale; MLNT: Multisyllabic Lexical Neighborhood Test; MSEL: The Mullen Scale of Early Learning; MUSS: Meaningful Use of Speech Scale; NVCQ: nonverbal cognitive quotient; PAQL: Paediatric Audiology Quality of Life questionnaire; PEDI: Pediatric Evaluation of Disability Inventory; PKB: Phonetically Balanced Kindergarten test; PLS: Preschool Language Scale; PLS-5: Preschool Language Scale-5; PSI: Parental Stress Index; RDLS: Reynell Developmental Language Scales; RGDS: Revised Gesell Developmental Schedules; SELSI: Sequenced Language Scale for Infants; SIR: Speech Intelligibility Rating; SRS: Geers and Moog Speech Reception Score; VABS: Vineland Adaptative Behaviour Scale Ed II; VSMS: Vineland Social Maturity Scale.

## Data Availability

All article lists are provided as Supplementary Material.

## Supplementary Materials

The following supporting information can be downloaded at: https://www.mdpi.com/article/10.3390/children10101653/s1, Table S1: List of assessment tools used in the 61 studies selected for our systematic review and their primary references. Reference List S1: Articles selected by Stage 1, listed in chronological order. Reference List S2: Articles selected by Stage 2, listed in chronological order. Reference List S3: Articles identified searching reference lists from articles selected in Stages 1 and 2, or in review articles, listed in chronological order.
