# Supplementary material for: Cochlear Implantation in Children with Additional Disabilities: A Systematic Review
Source: Children (Basel). 2023 Oct 5;10(10):1653. doi: 10.3390/children10101653 (PMC10605071; doi:10.3390/children10101653)
Supplement: Supplementary file 1 [file children-10-01653-s001.zip › children-2581963-supplementary.pdf]

Supplementary Material

Cochlear Implantation in Children with Additional Disabilities: A Systematic Review

Valeria Caragli [1], Daniele Monzani [2], Elisabetta Genovese [3], Silvia Palma [4] and Antonio M. Persico [5,*]

[1] Otorhinolaryngology-Head and Neck Surgery, Audiology Program, Department of Diagnostic Clinical and Public Health, University of Modena and Reggio Emilia, 41125 Modena, Italy; 196382@studenti.unimore.it
[2] Department of Surgery Dentistry Paediatrics and Gynaecology, University of Verona, 37100 Verona, Italy; daniele.monzani@univr.it
[3] Audiology Program, Department of Diagnostic Clinical and Public Health, University of Modena and Reggio Emilia, 41100 Modena, Italy; elisabetta.genovese@unimore.it
[4] Audiology, Primary Care Department, AUSL Modena, 41100 Modena, Italy; si.palma@ausl.mo.it
[5] Child and Adolescent Neuropsychiatry Program, Department of Biomedical, Metabolic and Neural Sciences, University of Modena and Reggio Emilia, Modena University Hospital, 41125 Modena, Italy
* Correspondence: antonio.persico@unimore.it

Suppl. Reference List S1: Articles selected by Stage 1, listed in chronological order and with their [num] as present in the main article reference list.

1. Amirsalari S, Yousefi J, Radfar S, Saburi A, Tavallaie SA, Hosseini MJ, Noohi S, Hassan Alifard M, Ajallouyean M. Cochlear implant outcomes in children with motor developmental delay. Int J Pediatr Otorhinolaryngol. 2012 Jan;76(1):100-3. doi: 10.1016/j.ijporl.2011.10.011. Epub 2011 Nov 17. PMID: 22100224. [15]
2. Boons T, Brokx JP, Dhooge I, Frijns JH, Peeraer L, Vermeulen A, Wouters J, van Wieringen A. Predictors of spoken language development following pediatric cochlear implantation. Ear Hear. 2012 Sep-Oct;33(5):617-39. doi: 10.1097/AUD.0b013e3182503e47. PMID: 22555184. [35]
3. Rafferty A, Martin J, Strachan D, Raine C. Cochlear implantation in children with complex needs - outcomes. Cochlear Implants Int. 2013 Mar;14(2):61-6. doi: 10.1179/1754762810Y.0000000009. Epub 2011 Jun 29. PMID: 22333941. [20]
4. Wakil N, Fitzpatrick EM, Olds J, Schramm D, Whittingham J. Long-term outcome after cochlear implantation in children with additional developmental disabilities. Int J Audiol. 2014 Sep;53(9):587-94. doi: 10.3109/14992027.2014.905716. Epub 2014 May 14. PMID: 24825367. [60]
5. van Nierop JW, Snabel RR, Langereis M, Pennings RJ, Admiraal RJ, Mylanus EA, Kunst HP. Paediatric Cochlear Implantation in Patients with Waardenburg Syndrome. Audiol Neurootol. 2016;21(3):187-94. doi: 10.1159/000444120. Epub 2016 Jun 1. PMID: 27245679; PMCID: PMC5296886. [32]
6. Trevisi P, Ciorba A, Aimoni C, Bovo R, Martini A. Outcomes of long-term audiological rehabilitation in charge syndrome. Acta Otorhinolaryngol Ital. 2016 Jun;36(3):206-14. doi: 10.14639/0392-100X-837. PMID: 27214832; PMCID: PMC4977008. [59]
7. Lachowska M, Pastuszka A, Łukaszewicz-Moszyńska Z, Mikołajewska L, Niemczyk K. Cochlear implantation in autistic children with profound sensorineural hearing loss. Braz J Otorhinolaryngol. 2016 Nov 19:S1808-8694(16)30231-2. doi: 10.1016/j.bjorl.2016.10.012. Epub ahead of print. PMID: 27939998. [62]
8. Zaidman-Zait A, Curle D, Jamieson JR, Chia R, Kozak FK. Health-Related Quality of Life Among Young Children With Cochlear Implants and Developmental Disabilities. Ear Hear. 2017 Jul/Aug;38(4):399-408. doi: 10.1097/AUD.0000000000000410. PMID: 28207575. [30]
9. Alzhrani F, Alhussini R, Hudeib R, et al. The outcome of cochlear implantation among children with genetic syndromes. European Archives of Oto-rhino-laryngology: Official Journal of the European Federation of Oto-rhino-laryngological Societies (EUFOS): Affiliated with the German Society for Oto-rhino-laryngology - Head and Neck Surgery. 2018 Feb;275(2):365-369. DOI: 10.1007/s00405-017-4832-0. PMID: 29204917. [41]
10. Vincenti V, Di Lella F, Falcioni M, Negri M, Zanetti D. Cochlear implantation in children with CHARGE syndrome: a report of eight cases. Eur Arch Otorhinolaryngol. 2018 Aug;275(8):1987-1993. doi: 10.1007/s00405-018-5053-x. Epub 2018 Jul 3. PMID: 29971494. [51]
11. Mesallam TA, Yousef M, Almasaad A. Auditory and language skills development after cochlear implantation in children with multiple disabilities. Eur Arch Otorhinolaryngol. 2019 Jan;276(1):49-55. doi: 10.1007/s00405-018-5184-0. Epub 2018 Oct 27. PMID: 30368552. [7]
12. Heldahl MG, Eksveen B, Bunne M. Cochlear implants in eight children with Down Syndrome - Auditory performance and challenges in assessment. Int J Pediatr Otorhinolaryngol. 2019 Nov; 126:109636. doi: 10.1016/j.ijporl.2019.109636. Epub 2019 Aug 12. PMID: 31442869. [63]
13. Datta G, Durbin K, Odell A, Ramirez-Inscoe J, Twomey T. An analysis of the five year outcomes of a cohort of 46 deaf children with severe (SLD) or profound and multiple learning difficulties (PMLD) and associated complex needs, including autism (ASD), tracked using the Nottingham Early Cognitive and Listening Links (Early CaLL): This framework monitors the relationship between sound processor use and listening, spoken language, cognition and communicative development, following cochlear

implantation. Cochlear Implants Int. 2020 Jan;21(1):35-45. doi: 10.1080/14670100.2019.1662587. Epub 2019 Sep 12. PMID: 31514587. [50]

14. Nair G, Dham R, Sekhar A, Kumar RS, Kameswaran M. Cochlear Implantation in Children with Usher's Syndrome: A South Asian Experience. Indian J Otolaryngol Head Neck Surg. 2020 Mar;72(1):140-144. doi: 10.1007/s12070-019-01759-y. Epub 2019 Nov 7. PMID: 32158671; PMCID: PMC7040150. [43]
15. Choo OS, Kim H, Kim YJ, Roh J, Jang JH, Park HY, Choung YH. Effect of Age at Cochlear Implantation in Educational Placement and Peer Relationships. Ear Hear. 2021 July/Aug;42(4):1054-1061. doi: 10.1097/AUD.0000000000001000. PMID: 33974787. [33]
16. Glaubitz C, Liebscher T, Hoppe U. Children with cochlear implant and additional disabilities benefit from consistent device use. Int J Pediatr Otorhinolaryngol. 2022 Nov; 162:111301. doi: 10.1016/j.ijporl.2022.111301. Epub 2022 Sep 6. PMID: 36096038. [73]

Suppl. Reference List S2: Articles selected by Stage 2, listed in chronological order and with their [num] as present in the main article reference list.

1. Smyth CM, Sinnathuray AR, Hughes AE, Toner JG. Cochlear implantation in keratitis-ichthyosis-deafness syndrome: 10-year follow-up of two patients. Cochlear Implants Int. 2012 Feb;13(1):54-9. doi: 10.1179/146701011X12950038111936. PMID: 22340753. [67]
2. Beer J, Harris MS, Kronenberger WG, Holt RF, Pisoni DB. Auditory skills, language development, and adaptive behavior of children with cochlear implants and additional disabilities. Int J Audiol. 2012 Jun;51(6):491-8. doi: 10.3109/14992027.2012.664291. Epub 2012 Apr 17. PMID: 22509948; PMCID: PMC3540405. [16]
3. Edwards L, Hill T, Mahon M. Quality of life in children and adolescents with cochlear implants and additional needs. Int J Pediatr Otorhinolaryngol. 2012 Jun;76(6):851-7. doi: 10.1016/j.ijporl.2012.02.057. Epub 2012 Mar 22. PMID: 22444739. [45]
4. Wiley S, Meinzen-Derr J, Grether S, Choo D, Hughes M. Longitudinal functional performance among children with cochlear implants and disabilities: A prospective study using the Pediatric Evaluation of Disability Inventory. International journal of pediatric otorhinolaryngology. 2012 76. 693-7. 10.1016/j.ijporl.2012.02.022. [11]
5. El Bakkouri W, Loundon N, Thierry B, Nevoux J, Marlin S, Rouillon I, Garabédian EN. Cochlear implantation and congenital deafness: perceptive and lexical results in 2 genetically pediatric identified population. Otol Neurotol. 2012 Jun;33(4):539-44. doi: 10.1097/MAO.0b013e31824bae35. PMID: 22569142. [37]
6. Cruz I, Vicaria I, Wang NY, Niparko J, Quittner AL; CDaCI Investigative Team. Language and behavioral outcomes in children with developmental disabilities using cochlear implants. Otol Neurotol. 2012 Jul;33(5):751-60. doi: 10.1097/MAO.0b013e3182595309. PMID: 22699986; PMCID: PMC3408892. [17]
7. Oghalai JS, Caudle SE, Bentley B, Abaya H, Lin J, Baker D, Emery C, Bortfeld H, Winzelberg J. Cognitive outcomes and familial stress after cochlear implantation in deaf children with and without developmental delays. Otol Neurotol. 2012 Aug;33(6):947-56. doi: 10.1097/MAO.0b013e318259b72b. PMID: 22710555; PMCID: PMC3399955. [18]
8. Youm HY, Moon IJ, Kim EY, Kim BY, Cho YS, Chung WH, Hong SH. The auditory and speech performance of children with intellectual disability after cochlear implantation. Acta Otolaryngol. 2013 Jan;133(1):59-69. doi: 10.3109/00016489.2012.720031. Epub 2012 Oct 15. PMID: 23066719. [21]
9. Birman CS, Elliott EJ, Gibson WP. Pediatric cochlear implants: additional disabilities prevalence, risk factors, and effect on language outcomes. Otol Neurotol. 2012 Oct;33(8):1347-52. doi: 10.1097/MAO.0b013e31826939cc. PMID: 22975903. [36]
10. Kontorinis G, Giesemann AM, Iliodromiti Z, Weidemann J, Aljeraisi T, Schwab B. Treating hearing loss in patients with infantile Bartter syndrome. Laryngoscope. 2012 Nov;122(11):2524-8. doi: 10.1002/lary.23532. Epub 2012 Sep 10. PMID: 22965860. [68]
11. Robertson J. Children with cochlear implants and autism – challenges and outcomes: The experience of the National Cochlear Implant Programme, Ireland. Cochlear Implants International. 2013 14. S11-S14. 10.1179/1467010013Z.000000000104. [47]
12. Jatana KR, Thomas D, Weber L, Mets MB, Silverman JB, Young NM. Usher syndrome: characteristics and outcomes of pediatric cochlear implant recipients. Otol Neurotol. 2013 Apr;34(3):484-9. doi: 10.1097/MAO.0b013e3182877ef2. PMID: 23442567. [72]
13. Byun H, Moon IJ, Kim EY, Park J, Kwon SY, Han HD, Chung WH, Cho YS, Hong SH. Performance after timely cochlear implantation in prelingually deaf children with cerebral palsy. Int J Pediatr Otorhinolaryngol. 2013 Jun;77(6):1013-8. doi: 10.1016/j.ijporl.2013.03.034. Epub 2013 Apr 29. PMID: 23639338. [19]

14. Ahn JH, Lee KS. Outcomes of cochlear implantation in children with CHARGE syndrome. Acta Otolaryngol. 2013 Nov;133(11):1148-53. doi: 10.3109/00016489.2013.814155. PMID: 24125186. [52]
15. Ricci G, Trabalzini F, Faralli M, D'Ascanio L, Cristi C, Molini E. Cochlear implantation in children with "CHARGE syndrome": surgical options and outcomes. Eur Arch Otorhinolaryngol. 2014 Mar;271(3):489-93. doi: 10.1007/s00405-013-2424-1. Epub 2013 Mar 24. PMID: 23525650. [55]
16. Birman CS, Brew JA, Gibson WP, Elliott EJ. CHARGE syndrome and Cochlear implantation: difficulties and outcomes in the paediatric population. Int J Pediatr Otorhinolaryngol. 2015 Apr;79(4):487-92. doi: 10.1016/j.ijporl.2015.01.004. Epub 2015 Jan 19. PMID: 25649713. [69]
17. Hilgenberg AM, Cardoso CC, Caldas FF, Tschiedel Rde S, Deperon TM, Bahmad F Jr. Hearing rehabilitation in cerebral palsy: development of language and hearing after cochlear implantation. Braz J Otorhinolaryngol. 2015 May-Jun;81(3):240-7. doi: 10.1016/j.bjorl.2014.10.002. Epub 2014 Oct 19. PMID: 25458256. [71]
18. dos Santos MJ, Lamônica DA, Ribeiro MV, McCracken W, Silva LT, Costa OA. Outcomes of cochlear implanted children with cerebral palsy: A holistic approach. Int J Pediatr Otorhinolaryngol. 2015 Jul;79(7):1090-5. doi: 10.1016/j.ijporl.2015.04.038. Epub 2015 May 6. PMID: 25977237. [54]
19. Eshraghi AA, Nazarian R, Telischi FF, Martinez D, Hodges A, Velandia S, Cejas-Cruz I, Balkany TJ, Lo K, Lang D. Cochlear Implantation in Children With Autism Spectrum Disorder. Otol Neurotol. 2015 Sep;36(8):e121-8. doi: 10.1097/MAO.0000000000000757. PMID: 25899551; PMCID: PMC4537326. [22]
20. Hashemi SB, Monshizadeh L. Comparison of Auditory Perception in Cochlear Implanted Children with and without Additional Disabilities. Iran J Med Sci. 2016 May;41(3):186-90. PMID: 27217602; PMCID: PMC4876296. [28]
21. Mikic B, Jotic A, Miric D, Nikolic M, Jankovic N, Arsovic N. Receptive speech in early implanted children later diagnosed with autism. Eur Ann Otorhinolaryngol Head Neck Dis. 2016 Jun;133 Suppl 1:S36-9. doi: 10.1016/j.anorl.2016.01.012. Epub 2016 May 27. PMID: 27246741. [39]
22. Valero MR, Sadadcharam M, Henderson L, Freeman SR, Lloyd S, Green KM, Bruce IA. Compliance with cochlear implantation in children subsequently diagnosed with autism spectrum disorder. Cochlear Implants Int. 2016 Jul;17(4):200-206. doi: 10.1080/14670100.2016.1211226. Epub 2016 Aug 4. PMID: 27491633. [70]
23. Hoshino AC, Echegoyen A, Goffi-Gomez MV, Tsuji RK, Bento RF. Outcomes of Late Implantation in Usher Syndrome Patients. Int Arch Otorhinolaryngol. 2017 Apr;21(2):140-143. doi: 10.1055/s-0036-1583306. Epub 2016 May 4. PMID: 28382120; PMCID: PMC5375700. [57]
24. Young NM, Tournis E, Sandy J, Hoff SR, Ryan M. Outcomes and Time to Emergence of Auditory Skills After Cochlear Implantation of Children With Charge Syndrome. Otol Neurotol. 2017 Sep;38(8):1085-1091. doi: 10.1097/MAO.0000000000001488. PMID: 28657954. [66]
25. Motegi M, Inagaki A, Minakata T, Sekiya S, Takahashi M, Sekiya Y, Murakami S. Developmental delays assessed using the Enjoji Scale in children with cochlear implants who have intellectual disability with or without autism spectrum disorder. Auris Nasus Larynx. 2019 Aug;46(4):498-506. doi: 10.1016/j.anl.2018.12.003. Epub 2018 Dec 19. PMID: 30579692. [23]
26. Aragón-Ramos P, Pedrero-Escalas MF, Gavilán J, Pérez-Mora R, Herrán-Martin B, Lassaletta L. Auditory Skills following Cochlear Implantation in Children with the Charge Syndrome. Audiol Neurootol. 2019;24(3):139-146. doi: 10.1159/000500659. Epub 2019 Jul 10. PMID: 31291620. [61]
27. Clarós P, Remjasz A, Clarós-Pujol A, Pujol C, Clarós A, Wiatrow A. Long-term Outcomes in Down Syndrome Children After Cochlear Implantation: Particular Issues and Considerations. Otol Neurotol. 2019 Dec;40(10):1278-1286. doi: 10.1097/MAO.0000000000002410. PMID: 31634275. [38]
28. Zhao Y, Li Y, Long Y, Jin X, Zheng Z, Liu Y, Wang Y, Zheng J, Zhang J, Chen M, Hao J, Yang Y, Liu W, Liu H, Ni X. Comparison of the development of early auditory and preverbal skills in Mandarin-Speaking children with cochlear implants with and without additional disabilities. Acta Otolaryngol. 2019 Dec;139(12):1098-1103. doi: 10.1080/00016489.2019.1670358. Epub 2019 Sep 27. PMID: 31560244. [29]

29. Omidvar S, Jeddi Z, Doosti A, Hashemi SB. Cochlear implant outcomes in children with attention-deficit/hyperactivity disorder: Comparison with controls. Int J Pediatr Otorhinolaryngol. 2020 Mar;130:109782. doi: 10.1016/j.ijporl.2019.109782. Epub 2019 Nov 16. PMID: 31785496. [42]
30. Friedmann DR, Tona KM, Roland JT Jr, Spitzer ER, Waltzman SB. Cochlear implantation in children under 12 months: Prevalence and implications of 'hidden' disabilities. Cochlear Implants Int. 2020 Nov;21(6):307-312. doi: 10.1080/14670100.2020.1773675. Epub 2020 Jun 7. PMID: 32508288. [64]
31. Micheletti S, Accorsi P, Giordano L, Calza S, Nassif N, Barezzani MG, Fazzi E, Redaelli de Zinis LO. Cognitive improvement after cochlear implantation in deaf children with associated disabilities. Dev Med Child Neurol. 2020 Dec;62(12):1429-1436. doi: 10.1111/dmcn.14671. Epub 2020 Sep 11. PMID: 32914885. [24]
32. Mancini P, Mariani L, Nicastri M, Cavicchiolo S, Giallini I, Scimemi P, Zanetti D, Montino S, Lovo E, Di Berardino F, Trevisi P, Santarelli R. Cochlear implantation in children with Autism Spectrum Disorder (ASD): Outcomes and implant fitting characteristics. Int J Pediatr Otorhinolaryngol. 2021 Oct;149:110876. doi: 10.1016/j.ijporl.2021.110876. Epub 2021 Aug 6. PMID: 34385039. [54]
33. Anne S, Schwartz SR, McCoy JL, Haberkamp T, Hoffer ME, Chi DH. Cochlear Implants in Neurologically Impaired Children: A Survey of Health-Related Quality of Life. Otolaryngol Head Neck Surg. 2021 Nov;165(5):731-738. doi: 10.1177/0194599821989642. Epub 2021 Feb 23. PMID: 33618581. [40]
34. Jenks CM, Hoff SR, Haney J, Tournis E, Thomas D, Young NM. Cochlear Implantation Can Improve Auditory Skills, Language and Social Engagement of Children With Autism Spectrum Disorder. Otol Neurotol. 2022 Mar 1;43(3):313-319. doi: 10.1097/MAO.0000000000003463. PMID: 34935761; PMCID: PMC8843363. [56]
35. Oghalai JS, Bortfeld H, Feldman HM, Chimalakonda N, Emery C, Choi JS, Zhou S. Cochlear Implants for Deaf Children With Early Developmental Impairment. Pediatrics. 2022 Jun 1;149(6):e2021055459. doi: 10.1542/peds.2021-055459. PMID: 35607935. [26]
36. Kay-Rivest E, McMenomey SO, Jethanamest D, Thomas Roland J Jr, Shapiro WH, Waltzman SB, Friedmann DR. Cochlear Implant Outcomes in CHARGE Syndrome: Updated Perspectives. Otol Neurotol. 2022 Jul 1;43(6):632-637. doi: 10.1097/MAO.0000000000003533. PMID: 35261375. [48]
37. Fan W, Ni K, Chen F, Li X. Hearing characteristics and cochlear implant effects in children with Waardenburg syndrome: a case series. Transl Pediatr 2022;11(7):1234-1241. doi: 10.21037/tp-22-271. [58]
38. Monshizadeh L, Hashemi SB, Rahimi M, Mohammadi M. Cochlear implantation outcomes in children with global developmental delay. Int J Pediatr Otorhinolaryngol. 2022 Nov;162:111213. doi: 10.1016/j.ijporl.2022.111213. Epub 2022 Jun 28. PMID: 35988456. [27]
39. Remjasz-Jurek A, Clarós P, Clarós-Pujol A, Pujol C, Clarós A. Outcomes of cochlear implantation in children with Usher syndrome: a long-term observation. Eur Arch Otorhinolaryngol. 2023 May;280(5):2119-2132. doi: 10.1007/s00405-022-07670-7. Epub 2022 Oct 15. PMID: 36242610. [44]

Suppl. Reference List S3: Articles identified searching reference lists from articles selected in Stages 1 and 2, or in review articles, listed in chronological order and with their [num] as present in the main article reference list.

1. Palmieri M, Berrettini S, Forli F, Trevisi P, Genovese E, Chilosi AM, Arslan E, Martini A. Evaluating benefits of cochlear implantation in deaf children with additional disabilities. Ear Hear. 2012 Nov-Dec;33(6):721-30. doi: 10.1097/AUD.0b013e31825b1a69. PMID: 22785571. [49]
2. Bunne M, The Oslo experience of cochlear implant in children with complex disorders and auditory neuropathy spectrum disorder, Cochlear Implants International, 2013 14:sup3, S31-S34, DOI: 10.1179/1467010013Z.000000000111. [34]
3. Broomfield SJ, Bruce IA, Henderson L, Ramsden RT, Green KM. Cochlear implantation in children with syndromic deafness. Int J Pediatr Otorhinolaryngol. 2013 Aug;77(8):1312-6. doi: 10.1016/j.ijporl.2013.05.022. Epub 2013 Jun 15. PMID: 23773333. [65]
4. Speaker RB, Roberston J, Simoes-Franklin C, Glynn F, Walshe P, Viani L. Quality of life outcomes in cochlear implantation of children with profound and multiple learning disability. Cochlear Implants Int. 2018 May;19(3):162-166. doi: 10.1080/14670100.2018.1434451. PMID: 29457562. [46]
5. Ganesh V, Ram B, Nandhan R, Kameswaran M. A Retrospective Clinical Audit of Outcomes of Cochlear Implantation in Children with Multiple Disabilities in Comparison with Normal Implantees: A South Indian Experience. Indian J Otolaryngol Head Neck Surg. 2021 Jun;73(2):140-146. doi: 10.1007/s12070-020-01844-7. Epub 2020 Apr 13. PMID: 34150587; PMCID: PMC8163895. [25]
6. Wiseman, Kathryn B.1; Warner-Czyz, Andrea D.1; Kwon, Shari1,2; Fiorentino, Kim1,2; Sweeney, Melissa1,2. Relationships Between Daily Device Use and Early Communication Outcomes in Young Children With Cochlear Implants. Ear and Hearing: July/August 2021 - Volume 42 - Issue 4 - p 1042-1053 doi: 10.1097/AUD.0000000000000999. [31]

Supplementary Table S1: List of assessment tools used in the 61 studies selected in our systematic review and their primary reference.

Outcome Measure	Reference
Alternative Scale (parents observation)	Killan CF, Baxter PD, Killan EC. Face and content validity analysis of the Speech, Spatial and Qualities of Hearing Scale for Parents (SSQ-P) when used in a clinical service without interviews or week-long observation periods. Int J Pediatr Otorhinolaryngol. 2020 Jun; 133:109964. doi: 10.1016/j.ijporl.2020.109964. PMID: 32114313.
ASQ-3: Ages and Stages questionnaire third edition	Vameghi R, Sajedi F, Kraskian Mojembari A, Habiollahi A, Lornezhad HR, Delavar B. Cross-Cultural Adaptation, Validation and Standardization of Ages and Stages Questionnaire (ASQ) in Iranian Children. Iran J Public Health. 2013 May 1; 42(5):522-8. PMID: 23802111; PMCID: PMC3684462.
Auditory Skills Checklist (ASC)	Meinzen-Derr J, Wiley S, Creighton J, Choo D. Auditory Skills Checklist: clinical tool for monitoring functional auditory skill development in young children with cochlear implants. Ann Otol Rhinol Laryngol. 2007 Nov; 116(11):812-8. doi: 10.1177/000348940711601104. PMID: 18074665.
Bayley Scale of Infant and Toddler Development, 3rd Edition (Bayley, III)	Bayley N (2006). Bayley scales of infant and toddler development, third edition: Administration Manual. San Antonio, TX: Harcourt.
Bench Kowal Bamford (BKB) Speech reception score	Bench J, Kowal A, Bamford J. The BKB (Bamford-Kowal-Bench) sentence lists for partially-hearing children. Br J Audiol. 1979 Aug; 13(3):108-12. doi: 10.3109/03005367909078884. PMID: 486816.
Categories of Auditory Performance (CAP)	Archbold S, Lutman ME, Nikolopoulos T. Categories of auditory performance: inter-user reliability. Br J Audiol. 1998 Feb; 32(1):7-12. doi: 10.3109/03005364000000045. PMID: 9643302.
Categories of Language (CL)	Bevilacqua MC, Delgado EMC, Moret ALM (1996). Estudos de casos clínicos de crianças do Centro Educacional do Deficiencite Auditvo (CEDAU), do hospital de Pesquisa e Reabilitação de Lesões Lábio-Palatinas – USP. In: Costa OA, Bevilacqua MC, organizadores. Anais do XI Encontro Internacional de Audiologia; March 30 – April 2, 1996. Bauru, Brasil. p.187
Child behavior checklist (CBCL)	Achenbach TM (2000). Child Behavior Checklist. In AE Kazdin (Ed.), Encyclopedia of Psychology (Vol. 2, pp. 69–70). Oxford University Press.
CNC: Consonant-Nucleus-Consonant	Peterson GE, Lehiste I. Revised CNC lists for auditory tests. J Speech Hear Disord 1962; 27:62–70.
DADQ: Deafness and Additional Disabilities Questionnaire	Palmieri M, Berrettini S, Forli F, Trevisi P, Genovese E, Chilosi AM, Arslan E, Martini A. Evaluating benefits of cochlear implantation in deaf children with additional disabilities. Ear Hear. 2012 Nov-Dec; 33(6):721-30. doi: 10.1097/AUD.0b013e31825b1a69. PMID: 22785571.
DAYC: Developmental Assessment of Young Children	Voress JK, Maddox T (1998). Developmental Assessment of Young Children: DAYC. Austin, TX: Pro-ed.
Delgado Test: List of words extracted from the child's vocabulary	Delgado EMC, Bevilacqua MC (1999). List of words to evaluated the speech perception in deaf children, Pro´ -Fono 11:59–64.
Developmental Profile-3	Alpern, G. D. (2007). Developmental profile 3 (DP-3). Los Angeles, CA: Western Psychological Services.
Early Speech Perception test (ESP test)	Jerger, S., & Jerger, J. (1984). Pediatric speech intelligibility test. Auditec of St. Louis.
EarlyCaLL	Datta G, Durbin K, Odell A, Ramirez-Inscoe J, Twomey T. The development and implementation of the Nottingham early cognitive and listening links (Early CaLL); A framework designed to support

	expectation counselling and to monitor the progress, post cochlear implantation, of deaf children with severe (SLD) and profound and multiple learning difficulties (PMLD) and associated complex needs. Cochlear Implants Int. 2020 Jan;21(1):18-34. doi: 10.1080/14670100.2019.1662586. PMID: 31514706.
EARS: Evaluation of Auditory Responses to Speech	Allum JH, Greisiger R, Straubhaar S, Carpenter MG. Auditory perception and speech identification in children with cochlear implants tested with the EARS protocol. Br J Audiol. 2000 Oct;34(5):293–303.
ELFRA – Elternfragebogen für die Früherkennung von Risikokindern	Klaiber, S. (2007). Erprobung des ELFRA (Elternfragebogen für die Früherkennung von Risikokindern): Probleme bei der Anwendung des ELFRA-1 und des ELFRA-2 (Doctoral dissertation, lmu).
Enjoji Scale of Infant Analytical Development	Motegi M, Inagaki A, Minakata T, Sekiya S, Takahashi M, Sekiya Y, Murakami S. Developmental delays assessed using the Enjoji Scale in children with cochlear implants who have intellectual disability with or without autism spectrum disorder. Auris Nasus Larynx. 2019 Aug;46(4):498-506. doi: 10.1016/j.anl.2018.12.003. PMID: 30579692.
Enjoji Scale of Infant Analytical Development (Enjoji Scale)	Motegi M, Inagaki A, Minakata T, Sekiya S, Takahashi M, Sekiya Y, Murakami S. Developmental delays assessed using the Enjoji Scale in children with cochlear implants who have intellectual disability with or without autism spectrum disorder. Auris Nasus Larynx. 2019 Aug; 46(4):498-506. doi: 10.1016/j.anl.2018.12.003. PMID: 30579692.
Freiburg Monosyllabic Word Test	Feldmann H. 200 years testing hearing disorders with speech, 50 years German speech audiometry – a review. Laryngorhinootologie 2004; 83: 735–742.
GASS: Grid Analysis of Spontaneous Speech	Chilosi, A. M., Comparini, A., Scusa, M. F., Orazini, L., Forli, F., Cipriani, P., & Berrettini, S. (2013). A longitudinal study of lexical and grammar development in deaf Italian children provided with early cochlear implantation. Ear and Hearing, 34(3), e28-e37.
Glendonald Auditory Screening Procedure (GASP)	Silva BCS, Moret ALM, Silva LTDN, Costa OAD, Alvarenga KF, Silva-Comerlatto MPD. Glendonald Auditory Screening Procedure (GASP): clinical markers of the development of auditory recognition and comprehension abilities in children using cochlear implants. Codas. 2019 Aug 15; 31(4):e20180142. Portuguese, English. doi: 10.1590/2317-1782/20192018142. PMID: 31433038.
GMDS: Griffiths Mental Development Scales	Griffiths R, Huntley M. Griffiths Mental Development Scales (Revised). High Wycombe UK, Test Agency Limited, 1996.
GMFCS: Gross Motor Function Classification System	Morris C, Bartlett D. Gross Motor Function Classification System: impact and utility. Dev Med Child Neurol. 2004 Jan;46(1):60-5. doi: 10.1017/s0012162204000118. PMID: 14974650.
Hearing In Noise Test (HINT)	Nilsson M, Soli SD, Sullivan JA. Development of the Hearing in Noise Test for the measurement of speech reception thresholds in quiet and in noise. J Acoust Soc Am. 1994 Feb;95(2):1085-99. doi: 10.1121/1.408469. PMID: 8132902.
Hochmair Schulz Moser sentence test in quiet	Hochmair-Desoyer I, Schulz E, Moser L. The HSM sentence test as a tool for evaluating the speech understanding in noise of cochlear implant users. Am J Otol 1997;18:83
HROoL: Health related quality of life	Patrick, D. L., Deyo, R. A., Atlas, S. J., Singer, D. E., Chapin, A., & Keller, R. B. (1995). Assessing health-related quality of life in patients with sciatica. Spine, 20(17):1899-1908.
IOWA Matrix Level B closed set Sentence Test	O'Donoghue G.M., Nikolopoulos T.P., Archbold S.M., Tait M. 1998. Speech perception in children after cochlear implantation. Am J Otol. 19: 762–7.
Kaufman Assessment Battery for Children	Kaufman, A. S., O'Neal, M. R., Avant, A. H., & Long, S. W. (1987). Introduction to the Kaufman Assessment Battery for Children (K-ABC) for pediatric neuroclinicians. J Child Neurol. 2(1):3-16.

Korean version of Ling's stages (K-Ling)	Moon IJ, Kim EY, Chu H, Chung WH, Cho YS, Hong SH. A new measurement tool for speech development based on Ling's stages of speech acquisition in pediatric cochlear implant recipients. Int J Pediatr Otorhinolaryngol 2011;75: 495–9
Lexical Neiborhood Test (LNT)	Kant AR, Banik AA. The Use of Lexical Neighborhood Test (LNT) in the Assessment of Speech Recognition Performance of Cochlear Implantees with Normal and Malformed Cochlea. Indian J Otolaryngol Head Neck Surg. 2017 Sep;69(3):338-344. doi: 10.1007/s12070-017-1142-1. Epub 2017 May 2. PMID: 28929065; PMCID: PMC5581769.
Ling's sounds test	Scollie S, Glista D, Tenhaaf J, Dunn A, Malandrino A, Keene K, Folkeard P. Stimuli and normative data for detection of Ling-6 sounds in hearing level. Am J Audiol. 2012 Dec;21(2):232-41. doi: 10.1044/1059-0889(2012/12-0020). Epub 2012 Jul 30. PMID: 22846636.
LIPS-R: Leiter International Performance Scale-Revised	Leiter RG (2002). Leiter international performance scale revised (LIPS-R). Western Psychological Service Publisher, Los Angeles, 12-187.
Listening progress score (LiP)	Nikolopoulos, T. P., Wells, P., & Archbold, S. M. (2000). Using Listening Progress Profile (LIP) to assess early functional auditory performance in young implanted children. Deafness & Education International, 2(3):142-151.
Little EARS	Coninx F, Weichbold V, Tsiakpini L, Autrique E, Bescond G, Tamas L, Compernol A, Georgescu M, Koroleva I, Le Maner-Idrissi G, Liang W, Madell J, Mikić B, Obrycka A, Pankowska A, Pascu A, Popescu R, Radulescu L, Rauhamäki T, Rouev P, Kabatova Z, Spitzer J, Thodi Ch, Varzic F, Vischer M, Wang L, Zavala JS, Brachmaier J. Validation of the LittlEARS((R)) Auditory Questionnaire in children with normal hearing. Int J Pediatr Otorhinolaryngol. 2009 Dec;73(12):1761-8. doi: 10.1016/j.ijporl.2009.09.036. PMID: 19836842.
MacArthur-Bates Communicative Development Inventories (CDI)	Fenson L, Marchman VA, Thal DJ, Dale PS, Reznick JS, Bates E. (2007) MacArthur-Bates communicative development inventories (2nd ed.). Baltimore: Paul H. Brookes.
Meaningful Auditory Integration Scale (MAIS/IT-MAIS)	Robbins AM, Renshaw JJ, Berry SW. Evaluating meaningful auditory integration in profoundly hearing-impaired children. Am J Otol. 1991;12 Suppl:144-50. PMID: 2069175.<br>Zimmerman-Phillips S, Robbins AM, Osberger MJ. Assessing cochlear implant benefit in very young children. Ann Otol Rhinol Laryngol Suppl. 2000 Dec; 185:42-3. doi: 10.1177/0003489400109s1217. PMID: 11140998.
Meaningful Use of Speech Scale (MUSS)	Robbins AM, Renshaw JJ, Berry SW. Evaluating meaningful auditory integration in profoundly hearing-impaired children. Am J Otol. 1991;12 Suppl:144-50. PMID: 2069175.
MSEL: The Mullen Scales of Early Learning	Mullen EM (1995). Mullen scales of early learning (AGS ed.). Circle Pines, MN: American Guidance Service Inc.
Multisyllabic Lexical Neiborhood Test (MLNT)	Kant AR, Banik AA. The Use of Lexical Neighborhood Test (LNT) in the Assessment of Speech Recognition Performance of Cochlear Implantees with Normal and Malformed Cochlea. Indian J Otolaryngol Head Neck Surg. 2017 Sep;69(3):338-344. doi: 10.1007/s12070-017-1142-1. PMID: 28929065; PMCID: PMC5581769.
NDS: Newsha Developmental Scale	Jafari Z, Asad-Malayeri S. The psychometric properties of newsha developmental scale: an integrated test for persian speaking children. Iran J Pediatr. 2012 Mar;22(1):28-34. PMID: 23056856; PMCID: PMC3448212.
NVCQ: nonverbal cognitive quotient	Meinzen-Derr J, Wiley S, Grether S, Choo DI. (2010). Language performance in children with cochlear implants and additional disabilities. Laryngoscope 2010; 120(2):405-413.
Oral and Written Language Scales, II Ed	Carrow-Woolfolk E (1995). Oral and written language scales (Vol. 93). Circle Pines, MN: American Guidance Service.

Peabody Picture Vocabulary Test (PPVT-4)	Dunn LM, Dunn DM (2007). Peabody picture vocabulary test, fourth edition: Manual. Minneapolis, MN: Pearson Assessments.
PEDI: Pediatric Evaluation of Disability Inventory	Dolva AS (1997). PEDI: Pediatric Evaluation of Disability Inventory. Ergoterapeuten 40(10): 20.
Pediatric Audiology Quality of life Questionnaire (PAQL)	Rajput K, Edwards L, Brock P, Abiodun A, Simpkin P, Al-Malky G. Ototoxicity-induced hearing loss and quality of life in survivors of paediatric cancer. Int J Pediatr Otorhinolaryngol. 2020 Nov; 138:110401. doi: 10.1016/j.ijporl.2020.110401. PMID: 33152988.
Phonetically Balance Kindergarten Test (PBK)	Meyer TA, Pisoni DB. Some computational analyses of the PBK test: effects of frequency and lexical density on spoken word recognition. Ear Hear. 1999 Aug;20(4):363-71. doi: 10.1097/00003446-199908000-00008. PMID: 10466571; PMCID: PMC3466479.
PLS: Preschool Language Scale	Zimmerman IL, Steiner VG, Pond ER (2011). Preschool language scale (5th ed.). San Antonio: Pearson.
Preschool Language Scale (PLS-4)	Zimmerman IL, Castilleja NF. The role of a language scale for infant and preschool assessment. Ment Retard Dev Disabil Res Rev. 2005;11(3):238-46. doi: 10.1002/mrdd.20078. PMID: 16161089.
Preschool Language Scale (PLS-5)	Zimmerman IL, Steiner VG, Pond RE (2011). PLS-5: Preschool language scale-5 [measurement instrument]. San Antonio, TX: Psychological Corporation.
PSI: Parental Stress Index	Abidin R, Flens JR, Austin WG. (2006). The parenting stress index. Lawrence Erlbaum Associates Publishers. In RP Archer (Ed.), Forensic uses of clinical assessment instruments (pp. 297–328). Lawrence Erlbaum Associates Publishers. https://doi.org/10.1037/14643-02
PVEIQ questionnaire	Anne S, Schwartz SR, McCoy JL, Haberkamp T, Hoffer ME, Chi DH. (2021). Cochlear implants in neurologically impaired children: a survey of health-related quality of life. Otolaryngol Head Neck Surg. 2021 Nov;165(5):731-738. doi: 10.1177/0194599821989642. Epub 2021 Feb 23. PMID: 33618581.
PVEIQ questionnaire	Nicholas JG, Geers AE. Personal, social, and family adjustment in school-aged children with a cochlear implant. Ear Hear. 2003;24:69S-81S.
Questionnaire for Measuring Health-Related Quality of Life in Children and Adolescents (KINDL)	Villalonga-Olives E, Kiese-Himmel C, Witte C, Almansa J, Dusilova I, Hacker K, von Steinbuechel N. Self-reported health-related quality of life in kindergarten children: psychometric properties of the Kiddy-KINDL. Public Health. 2015 Jul;129(7):889-95. doi: 10.1016/j.puhe.2015.04.020. Epub 2015 Jun 4. PMID: 26051961.
Receptive-Expressive Emergent Language Test III Ed	Bzoch KR, League R, Brown VL (2003). Receptive-expressive Emergent Language Test: Examiner's Manual. Austin: Pro-ed.
Reynell Developmental Language Scale (RDLS)	Reynell J, Gruber C (1990). Reynell developmental language scales. Los Angeles: Western Psychological Services.
Schlichting Expressive Language Test (SELT)	Boons T, Brokx JP, Frijns JH, Peeraer L, Philips B, Vermeulen A, Wouters J, van Wieringen A. Effect of pediatric bilateral cochlear implantation on language development. Arch Pediatr Adolesc Med. 2012 Jan; 166(1):28-34. doi: 10.1001/archpediatrics.2011.748. PMID: 22213747.
Sequenced language scale for infant (SELSI)	Kim YT (2002). Content and reliability analyses of the Sequenced Language Scale for Infants (SELSI). Commun Sci Disord. 2002; 7:1–23.
Speech Intelligibility Ratings (SIR)	Cox RM, McDaniel DM. Development of the Speech Intelligibility Rating (SIR) test for hearing aid comparisons. J Speech Hear Res. 1989 Jun;32(2):347-52. doi: 10.1044/jshr.3202.347. PMID: 2739387.
Speech perception abilities (Geers and Moog)	Geers AE, Moog JS. Speech perception and production skills of students with impaired hearing from oral and total communication education settings. J Speech Hear Res. 1992 Dec;35(6):1384-93. doi: 10.1044/jshr.3506.1384. PMID: 1494280.

Speech Recognition Index in Quiet (SRIQ)	Wang NY, Eisenberg LS, Johnson KC, Fink NE, Tobey EA, Quittner AL, Niparko JK; CDaCI Investigative Team. Tracking development of speech recognition: longitudinal data from hierarchical assessments in the Childhood Development after Cochlear Implantation Study. Otol Neurotol. 2008 Feb; 29(2):240-5. doi: 10.1097/MAO.0b013e3181627a37. PMID: 18223451; PMCID: PMC2733235.
Spondee test	Schlauch RS, Han HJ, Yu TJ, Carney E. Pure-Tone-Spondee Threshold Relationships in Functional Hearing Loss: A Test of Loudness Contribution. J Speech Lang Hear Res. 2017 Jan 1; 60(1):136-143. doi: 10.1044/2016_JSLHR-H-15-0330. PMID: 27973669.
SRIQ: Speech Recognition Index in Quiet	Wiseman KB, Warner-Czyz AD (2018). Adapted Speech Recognition Index in Quiet (SRI-Q) [Database record]. APA PsycTests. https://doi.org/10.1037/t72829-000.
Tanaka-Binet test	Uno Y, Mizukami H, Ando M, Yukihiro R, Iwasaki Y, Ozaki N. Reliability and validity of the new Tanaka B Intelligence Scale scores: a group intelligence test. PLoS One. 2014 Jun 18;9(6):e100262. doi: 10.1371/journal.pone.0100262. PMID: 24940880; PMCID: PMC4062530.
Tanaka–Binet Test	Kurita H, Uchiyama T, Takesada M (1985). Tokyo Child Development Schedule—I. Test-Retest Reliability and Concurrent Validity. Folia Psychiatr Neurol Jpn. 1985; 39(2):129-37. doi: 10.1111/j.1440-1819.1985.tb02896.x. PMID: 4065757.
Test for Reception of Grammar (TROG)	Bishop DV (1983). Test for reception of grammar. Published by the author and available from Age and Cognitive Performance Research Centre, University of Manchester, M13 9PL.
TIPI1, TIPI2 (Test identificazione parole infantili 1 e 2)	Arslan E, Genovese E, Orzan E, Turrini M (1997). Valutazione della percezione verbale nel bambino ipoacusico: Test di Identificazione di Parole Infantili 1–2 (Evaluation of verbal perception in children with hearing loss: Infant Words Identification Test 1–2). Bari, Italy: Ecumenica.
VABS: Vineland Adaptative Behaviour Scale	Sparrow SS, Cicchetti DV (1989). The Vineland adaptive behavior scales. In CS Newmark (Ed.), Major psychological assessment instruments, Vol. 2, pp. 199–231). Boston, London: Allyn & Bacon.
VSMS: Vineland Social Maturity Scale	Pedrini DT, Pedrini BC (1966). The vineland social maturity scale: Recommendations for administration, scoring and analysis. J Sch Psychol 5(1):14-20. ISSN 0022-4405, https://doi.org/10.1016/0022-4405(66)90083-5.
Weschler Intelligence Scale	Wechsler D (1949). Wechsler intelligence scale for children (Vol. 1). New York: Psychological Corporation.
Weschler Preschool and Primary Scale of Intelligence-III	Wechsler D (2002). WPPSI-III technical and interpretive manual. San Antonio, TX: The Psychological Corporation.
